# Fixed-Bed Bioreactor Culture Enhances Yield and Reparative Properties of hTERT Mesenchymal Stem Cell Extracellular Vesicles

**DOI:** 10.3390/cells15070654

**Published:** 2026-04-07

**Authors:** Zachary Cuba, Lenny Godinho, Sujata Choudhury, Kajal Patil, Anastasia Williams, Weidong Zhou, Marissa Howard, Surya P. Aryal, Kevin A. Clayton, David A. Routenberg, Lance A. Liotta, Heather Couch, Fatah Kashanchi, Heather Branscome

**Affiliations:** 1 American Type Culture Collection, Manassas, VA 20110, USA; 2 Laboratory of Molecular Virology, School of Systems Biology, George Mason University, Manassas, VA 20110, USA; 3 Center for Applied Proteomics and Molecular Medicine, George Mason University, Manassas, VA 20110, USA; 4 Meso Scale Diagnostics, LLC, Rockville, MD 20850, USA

**Keywords:** extracellular vesicles, hTERT, mesenchymal stem cells, fixed-bed bioreactor

## Abstract

Mesenchymal stem cells (MSCs) are multipotent cells that have the ability to mediate cellular repair through a combination of soluble paracrine factors, as well as bioactive cargo packaged within extracellular vesicles (EVs). Although MSC-derived EVs have been widely investigated for their regenerative potential, progress toward translational evaluation has been limited in part by challenges in scalable and reproducible manufacturing. We recently reported that human telomerase reverse transcriptase (hTERT)-immortalized MSCs reproducibly produce EVs that retain key characteristics of EVs derived from primary MSCs. Building on this work, three-dimensional (3D) culture systems have emerged as promising platforms for large-scale manufacturing. In this study, we compared the yield, molecular composition, and functional activity of EVs produced from hTERT-immortalized MSCs cultured in either a fixed-bed bioreactor or conventional two-dimensional (2D) flasks. Our data demonstrate that bioreactor culture results in increased EV yield as compared to an equivalent production from 2D cultures. Molecular analyses indicated that bioreactor-derived EVs were associated with a broader spectrum of cargo and were enriched with molecules that may contribute to enhanced reparative function. Importantly, bioreactor-derived EVs also exerted a more pronounced effect in cellular repair assays in vitro. Collectively, these results highlight the potential of fixed-bed bioreactors as scalable platforms for EV production, offering higher yields while preserving molecular composition and functional activity. This approach represents an important step toward achieving the reproducible, high-quality EV production required for research and future translational applications.

## 1. Introduction

Mesenchymal stem cells (MSCs), also known as mesenchymal stromal cells or medicinal signaling cells, are a population of multipotent cells capable of exerting a range of reparative properties at the cellular and tissue levels. MSCs can be found in several mesoderm-derived tissue reservoirs, including bone marrow, peripheral blood, adipose, and dental pulp, as well as neonatal tissues such as the placenta, umbilical cord, cord blood, and Wharton’s Jelly [1,2,3,4]. The diversity of tissue sources from which MSCs can be isolated, coupled with their reparative properties, has contributed to their widespread research applications, particularly in the field of regenerative medicine. Along these lines, there are over 1600 studies registered on ClinicalTrials.gov referencing MSCs as interventions or treatments for various diseases and conditions.

Within the last two decades, it has been demonstrated that the therapeutic effects of MSCs are due to MSC-secreted paracrine factors; these factors have largely been confirmed to be contained and transported within extracellular vesicles (EVs) [5,6,7,8]. The term “EVs” refers to a diverse group of nano-sized particles, enclosed by lipid-membranes, that are released from all cells under both physiological and pathological conditions. Upon their discovery in the 1960s, EVs were originally considered to be merely cellular ‘junk’ or debris; however, in the last thirty years they have clearly been shown to play important roles in various processes such as cellular homeostasis, cell-to-cell communication, cell migration, and immune regulation [9,10,11]. In recent years, MSC EVs have been shown to carry a suite of bioactive cargo, including coding and non-coding RNAs, signaling molecules (e.g., cytokines, chemokines, and interleukins), and proteins associated with cellular repair and tissue remodeling [12,13]. Perhaps most importantly, MSC EVs offer several potential therapeutic advantages over their parental stem cells due to their lower immunogenicity, enhanced ability to cross biological barriers, greater stability, and increased potential to be engineered for targeted administration [14].

A recent systematic review reports that MSC-derived extracellular EVs have been evaluated in over 60 registered clinical studies, primarily targeting inflammatory, immune-mediated, and tissue repair indications including neurological injury, pulmonary disease, musculoskeletal disorders, and cutaneous wound healing [15]. Despite their high therapeutic potential, there are some challenges surrounding the use of MSC EVs. These include, but are not limited to, heterogeneity, scalability, reproducibility, and the lack of standardized manufacturing and quality control testing protocols [16,17,18]. For example, tissue source, media composition, and culture properties (e.g., 2D vs. 3D) are upstream factors that can contribute to variations in EV populations. Downstream factors, including EV isolation protocols, formulation, and storage conditions, also have the potential to significantly impact EV heterogeneity [19]. To address these challenges and to promote harmonization and standardization, international societies have proposed a set of minimal criteria for MSC EVs. Collectively, these criteria provide guidance throughout the EV isolation process and offer recommendations for defining the biochemical, physical, and functional properties of EVs [20]. Adherence to these guidelines will be critical for advancing MSC EV research and contributing to its future clinical translation.

Over the last few decades, advances in cell line engineering, namely protocols utilizing human telomerase reverse transcriptase (hTERT) immortalization, have provided an efficient and effective mechanism for extending the cellular lifespan and improving the stability of primary cells without significant adverse effects. Briefly, this technology involves engineering of primary cells to ectopically express the hTERT gene. Continuous expression of hTERT prevents telomere erosion during cell division, which, in turn, delays the onset of cellular senescence [21]. Thus, stable cultures of hTERT-immortalized cells can be further expanded over extended periods without undergoing replicative senescence. This enables the production of working cell banks that are well-suited for preclinical studies and regenerative medicine applications.

Two of the many advantages of using hTERT-immortalized MSCs are their reproducibility and consistency. For example, the use of these cells has the potential to temper some of the inherent variability associated with EV production from primary stem cells. Along these lines, our lab was among the first to report on the reproducible production of functional EVs from hTERT-immortalized MSCs [22]. Others have also recently reported that EVs derived from hTERT-immortalized MSCs retain many of the reparative properties observed in EVs from primary MSCs, including in vitro and in vivo immunomodulation, inflammation suppression, proliferation promotion, and fibrosis reduction [23,24,25,26,27].

As the field of EV research advances, there is a growing need to explore novel, robust, and scalable technologies to improve EV production capacity. Three-dimensional (3D) cell culture systems represent an advanced platform that enables cells to grow in a microenvironment that more closely mimics the in vivo conditions relative to planar/two-dimensional (2D) cultures. In general, 3D culture techniques can be classified as either scaffold-based or scaffold-free, with the former providing physical and mechanical support that promotes adherent cell growth and the latter relying on techniques that promote suspended cell growth in the absence of a scaffold [28]. Although a comprehensive overview of the benefits and drawbacks of each of these systems is outside the scope of the current manuscript, it is worth noting that different 3D culture systems have recently been explored for culturing MSCs at-scale. These studies have suggested that EVs isolated from 3D MSC cultures display increased yield while maintaining robust functional and reparative properties [29,30,31,32,33,34].

We have previously reported the large-scale, reproducible production of EVs from different stem cell sources, namely primary MSCs, induced pluripotent stem cells (iPSCs), and hTERT-immortalized MSCs, using 2D culture systems. In those studies, we demonstrated the reparative properties of the EVs across different cell types using both 2D and 3D in vitro models [22,35,36]. In the current manuscript, we evaluated a fixed-bed bioreactor system as a proof-of-concept platform for producing EVs from hTERT-immortalized MSCs (hereafter referred to as hTERT MSCs). This platform was originally developed for scalable, continuous, and high-capacity adherent cell-based production, leading us to rationalized that it could be effectively adapted for EV production. Importantly, the system provides continuous control and real-time monitoring of critical process parameters (e.g., temperature, oxygen, and pH) throughout production. To the best of our knowledge, we are among the first labs to publish proof-of-concept data supporting scalable EV production from MSCs using this fixed-bed bioreactor platform. Our data demonstrate that the fixed-bed bioreactor improves the growth kinetics of hTERT MSCs relative to 2D culture and results in increased EV yield as compared to EVs isolated from a comparable bulk volume (i.e., 5 L) collected from 2D flask-based cultures. Importantly, our data also suggests that hTERT MSC EVs produced from this platform exert functional effects in vitro to promote cell migration and immunomodulation. Collectively, these results highlight the potential of fix-bed bioreactors as novel, scalable platforms for the production of MSC EVs that retain key functional and reparative attributes.

## 2. Materials and Methods

### 2.1. Cell Lines

All cell lines were obtained from ATCC (Manassas, VA, USA). Human telomerase reverse transcriptase (hTERT) mesenchymal stem cells (MSCs) (ATCC^®^ SCRC-4000™) were maintained in Mesenchymal Stem Cell Basal Medium (ATCC^®^ PCS-500-030™) supplemented with Mesenchymal Stem Cell Growth Kit (ATCC^®^ PCS-500-040™). Normal human primary dermal fibroblasts (ATCC^®^ PCS-201-012™) were maintained in Fibroblast Basal Medium (ATCC^®^ PCS-201-030™) supplemented with Fibroblast Growth Kit (ATCC^®^ PCS-201-040™). Normal primary epidermal keratinocytes (ATCC^®^ PCS-200-011™) were maintained in Dermal Cell Basal Medium (ATCC^®^ PCS-200-030™) supplemented with Keratinocyte Growth Kit (ATCC^®^ PCS-200-040™). The human keratinocyte cell line Ker-CT (ATCC^®^ CRL-4048™) was maintained in KGM Gold Keratinocyte Growth Medium BulletKit (Lonza, Walkersville, MD, USA). All cell lines were cultured in accordance with the manufacturer’s guidelines.

### 2.2. hTERT MSC Expansion in the Fixed-Bed Bioreactor

hTERT MSCs were first expanded in multilayer vessels to approximately 2.0 × 10^9^ total viable cells before seeding the scale-X Hydro fixed-bed bioreactor (Univercells Technologies, Nivelles, Belgium). Prior to use, the bioreactor vessel, tubing manifold kit, and calibrated pH and dissolved oxygen probes were sterilized by autoclaving. The sterilized tubing assembly was connected to three sterile pharmatainer vessels: a 1 L sampling vessel for media and cell inoculation, a 1 L vessel for pressure stabilization, and a 5 L vessel for media recirculation. A heating jacket was installed around the bioreactor to maintain temperature at 37 °C, and a temperature probe was positioned into the thermowell located in the bioreactor lid. All probes were connected to the controller unit for real-time monitoring of process parameters.

Following setup, 700 mL of pre-warmed culture medium was pumped into the bioreactor from the sampling vessel. Once the system reached the target temperature, hTERT MSCs were inoculated into the bioreactor to seed the fixed bed (Day 0). In parallel, cells were seeded into T-25 cm^2^ flasks under identical conditions to serve as controls for growth and viability. After inoculation, the magnetic stirrer speed was set to 1 cm/s for approximately 3 h to facilitate uniform cell attachment. Subsequently, the stirrer speed was reduced to 0.5 cm/s, and media recirculation was initiated from the 5 L pharmatainer. Culture progress was monitored by tracking oxygen consumption, pH levels, and glucose concentration. Additionally, cell density and viability were periodically assessed by removing sampling strips for cell counting.

For cell counting, individual sampling strips were aseptically removed from the bioreactor at the corresponding time point. Sample strips were aseptically transferred to a sterile microcentrifuge tube, and cells were enzymatically detached to obtain a single cell suspension. Cells were trypsinized from a control flask in parallel. Cell number and viability were measured via trypan blue exclusion using the Vi-CELL BLU automated cell counter (Beckman Coulter, Brea, CA, USA). Prior to use, the machine was properly calibrated with the manufacturer’s 2M CC (Concentration Control) beads. All samples were analyzed in duplicate, with 100 images captured per replicate. To determine the cell yield, the total viable cell number was divided by the corresponding surface area of the sampling strip (12.5 cm^2^) or control flask (25 cm^2^).

### 2.3. EV Isolation

**Figure 1:**
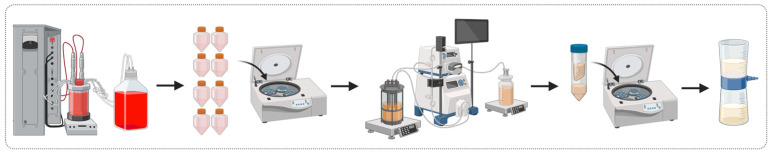
Overview of EV isolation workflow. hTERT MSCs are expanded in a fixed-bed bioreactor and EVs are harvested from conditioned culture medium by clarification, TFF-based concentration and diafiltration, centrifugal ultrafiltration, followed by sterile filtration. Figure created with BioRender.com (https://www.biorender.com, accessed 1 March 2026).

For this study, extracellular vesicles (EVs) were exclusively isolated from bioreactor cultures of hTERT MSCs. The flask-derived EVs used for comparative analyses were previously manufactured using the same downstream workflows and are equivalent to ATCC^®^ SCRC-4000-EXM™. Conditioned medium from each production platform was processed using an identical tangential flow filtration (TFF)-based method, following protocols described in our prior publications [22,35]. EV-depleted FBS was used in the culture medium prior to EV isolation. Conditioned medium was initially clarified by centrifugation at 2000× *g* to remove cells and large vesicles. The clarified supernatant was concentrated by TFF using the KrosFlo KR2i system with a 500 kDa molecular weight cut off (MWCO) filter (Repligen Corporation, Waltham, MA, USA). All runs were performed using the same membrane type, system configuration, and target operating ranges for feed flow, transmembrane pressure, and shear rate. Diafiltration was performed with PBS using a 5× volume exchange. Following TFF processing, EV preparations were further concentrated using centrifugal ultrafiltration and subsequently sterile-filtered through 0.2 µm membranes. Vialed aliquots were frozen at −20 °C. An overview of the EV isolation workflow is shown in Figure 1.

### 2.4. Nanoparticle Tracking Analysis

EV concentration (particles/mL) and size distribution were measured using the NanoSight NS300 nanoparticle tracking analysis (NTA) platform (Malvern Panalytical, Malvern, UK). Prior to NTA analysis, EVs were diluted in sterile PBS. Measurements were performed at room temperature using a syringe pump to maintain a uniform flow rate. Each sample was analyzed in duplicate, with three 30-s videos captured per replicate under controlled instrument settings. Raw data were processed with the NanoSight NTA software version 3.4. The machine is regularly maintained and serviced according to the manufacturer’s guidelines.

### 2.5. Multiplex Immunoassays for EV Surface Marker Profiling

Prior to surface marker profiling, EV samples were normalized based on particle concentration determined by NTA to ensure equivalent input across experiments. A rangefinding study was then performed using R-PLEX^®^ EV electrochemiluminescence assays (Meso Scale Diagnostics (MSD), Rockville, MD, USA) to estimate overall levels of tetraspanin-positive vesicles and guide the selection of starting dilutions for subsequent assays. Each sample was tested at two dilutions in triplicate using capture antibodies for CD63, CD81, and CD9 displayed on U-PLEX^®^ plates (MSD, Rockville, MD, USA). Detection was performed with a cocktail of electrochemiluminescent (ECL)-labeled antibodies targeting the same tetraspanins. The assay calibrator, a preparation of EVs from Expi293 cells engineered to express consistent levels of all three tetraspanins, was serially diluted to generate a standard curve. ECL signals from test samples were back-fitted to the standard curve to calculate concentration in Arbitrary Units (AU), where 1AU corresponds to the stock concentration of the calibrator blend. This approach enables accurate quantitative comparison of samples across a wide dynamic range and between multiple experiments.

For further EV profiling, custom ultrasensitive assay panels were configured to quantify EVs with additional surface markers including key MSC surface proteins. Capture antibodies were displayed on U-PLEX^®^ plates (MSD, Rockville, MD, USA) in three panels and each panel included an isotype control antibody to monitor nonspecific binding. Detection was performed using a cocktail of CD63, CD81 and CD9 antibodies with ultrasensitive detection labels. Based on range finding results and the wide expected range of expression among EV subpopulations, samples were assayed at three dilutions to confirm dilution linearity and ensure at least one dilution fell within the dynamic range of the assay. Each dilution was tested in triplicate. Plates were read on a SECTOR Imager in high dynamic range mode. Calibration curves for each panel were generated using a blend of EV calibrators derived from recombinant cell lines, each overexpressing one target antigen as well as the three tetraspanins, and concentrations were calculated in AU as described above for the R-PLEX^®^ assays. 

### 2.6. Western Blot

Protein levels were quantified using the Bicinchoninic Acid (BCA) assay. Equal amounts of EV protein or cell lysate protein were combined with Laemmli sample buffer and denatured at 95 °C. Proteins were resolved on 4–20% Tris–glycine polyacrylamide gels (Invitrogen, Carlsbad, CA, USA) and subsequently transferred to PVDF membranes using an overnight transfer protocol. Membranes were blocked and incubated with primary antibodies at 4 °C overnight. Primary antibodies included α-CD63, α-CD81, α-CD9, α-Flotillin-1, α-Alix, α-Actin, α-Cyclin A, α-Cyclin D1, α-Cyclin B1, α-Cdk2, α-Cdk6, α-GAPDH. After washing, membranes were incubated with the appropriate HRP-conjugated secondary antibodies and visualized using Clarity or Clarity Max chemiluminescent substrates (Bio-Rad, Hercules, CA, USA). Images were captured with the ChemiDoc Imaging System (Bio-Rad). Raw images of blots can be found in Figure S1.

### 2.7. Proteomics

Isolated EVs were subjected to proteomic profiling by liquid chromatography–tandem mass spectrometry (LC-MS/MS). Analyses were performed on an Exploris 480 instrument (Thermo Fisher Scientific, Waltham, MA, USA). Raw files were processed in Proteome Discoverer v2.4 against the NCBI human reference database. Peptide-spectrum matches were filtered using a 1% false discovery rate (FDR) to ensure high-confidence identifications; master proteins passing these criteria were carried forward for downstream analyses. To compare EV cargo, the master protein lists were intersected using InteractiVenn (https://www.interactivenn.net, accessed 10 December 2025). Functional relationships among identified proteins were evaluated using Search Tool for the Retrieval of Interacting Genes/Proteins (STRING) (https://string-db.org, accessed 10 December 2025). Networks were constructed with a combined confidence score ≥ 0.7 (high confidence) and disconnected nodes were removed before clustering. Protein interaction networks were clustered using the Markow Cluster Algorithm (MCA) with an inflation parameter of 2, which provides a balance between cluster specificity and inclusiveness. This approach groups proteins into biologically meaningful modules based on connectivity patterns within the STRING network. Clusters containing more than three proteins were retained for visualization and functional annotation. Functional enrichment was performed across multiple annotation systems (Gene Ontology (GO) Biological Process, Kyoto Encyclopedia of Genes and Genomes (KEGG) pathways, Reactome pathways). Bubble plots were generated from Reactome to illustrate the most significant processes. Bubble area represents the number of identified proteins mapped to each pathway (noted by Reactome as “gene count”) and color intensity reflects FDR-adjusted significance.

### 2.8. Cell Viability and Repair Assays

Primary human keratinocytes were seeded into 96-well plates overnight. Cells were then exposed to ionizing radiation (IR) at a dose of 2.5 Gy using an RS 2000 X-ray Irradiator (Rad Source Technologies, Buford, GA, USA). Immediately following irradiation (day 0), cultures were treated with hTERT MSC EVs at an approximate ratio of 1:10,000 (cell:EV ratio). Untreated controls received culture medium only. All conditions were performed in triplicate. Cell viability was assessed daily over a 72-h period using the CellTiter-Glo Luminescent Cell Viability Assay (Promega, Madison, WI, USA) and quantified on a GloMax Multi Detection System (Promega, Madison, WI, USA). Individual plates were read at 24, 48, and 72 h post-treatment.

### 2.9. Cell Migration Assay

Cells were seeded into cell culture inserts in complete growth medium and incubated overnight to allow attachment. Once cells reached full confluence, a gap of approximately 500 µm was introduced by carefully removing the inserts. Cultures were rinsed with PBS to remove debris, and basal (unsupplemented) media was added to each well. Experimental conditions included untreated controls (basal media only) and EV-treated groups, which received either hTERT MSC EVs (bioreactor) or PC-3 EVs (ATCC^®^ CRL-1435-EXM™) at a ratio of approximately 1:5000 (recipient cell:EV). Each sample was tested in duplicate. Cultures were incubated over a period of 72 h, and cell migration was imaged using the BioTek Cytation at multiple time points. Images were analyzed using ImageJ software (version 1.54d) with the Phantast plugin to quantify the percentage of gap closure over time [37].

### 2.10. RNA Sequencing

Total RNA was extracted from EVs using phenol-chloroform and RNA concentration was assessed using a NanoDrop One spectrophotometer (Thermo Fisher Scientific, Waltham, MA, USA). RNA sequencing and bioinformatic analysis were performed by CD Genomics (Shirley, NY, USA) following their standard protocols. Briefly, libraries were prepared from 0.01–1 μg RNA after rRNA depletion and fragmentation and were sequenced on an Illumina NovaSeq X Plus platform (paired-end 150 bp).

Raw reads in FASTQ format were quality-filtered to remove adapter sequences, reads with >10% ambiguous bases, and reads with >50% low-quality bases. Clean reads were aligned to the human reference genome (GRCh38, GENCODE) using HISAT2. Transcript assembly and quantification were performed with StringTie, and expression levels were calculated as Fragments Per Kilobase of transcript per Million mapped reads (FPKM) using Cufflinks components (Cuffquant and Cuffnorm). Differential expression analysis was conducted using DESeq2 (FDR ≤ 0.05, |log_2_FC| > 1). Functional annotation of differentially expressed genes employed GO and KEGG enrichment analyses. Long noncoding RNAs were identified based on transcript length (>200 nt), exon count (>2), and coding potential assessed by Coding Potential Calculator (CPC), Coding-Noncoding Index (CNCI), Protein families database (Pfam), and Coding-Potential Assessment Tool (CPAT).

### 2.11. Statistical Analysis

Statistical analyses were carried out using GraphPad Prism version 9.0. Depending on the experimental design, comparisons were made using an unpaired *t*-test, one-way ANOVA, or two-way ANOVA. Statistical significance was defined as follows: *p* < 0.05 (significant), *p* < 0.01 (highly significant), *p* < 0.001 (very highly significant), and *p* < 0.0001 (extremely significant).

## 3. Results

### 3.1. Fixed-Bed Bioreactor Culture Enhances hTERT MSC Expansion and Growth Kinetics

We have previously demonstrated the ability to isolate well-characterized and functional EVs from hTERT MSCs produced from 2D (e.g., CellSTACK multi-layer flasks/vessels) cultures. Although these platforms can be scaled to achieve multiple liters of conditioned media, they are labor-intensive, have a large physical footprint, and offer limited control over culture conditions. Bioreactors, particularly fixed-bed systems, are an attractive alternative due to their compact size, scalability, and ability to maintain controlled and reproducible culture conditions. For these reasons, we sought to evaluate a fixed-bed bioreactor as a scalable platform for the expansion of hTERT MSCs and to determine its relative efficiency for EV production. The fixed-bed bioreactor we used has approximately 24,000 cm^2^ surface area for cell attachment and growth and can accommodate production volumes up to 5 L. To achieve the number of cells required for seeding the bioreactor (≥2.0 × 10^8^ total viable cells), hTERT MSCs were initially cultured 2D flasks. On the day that the bioreactor was seeded (day 0), a set of control T-flasks were also seeded in parallel under the same conditions to compare cell growth throughout expansion.

**Figure 2:**
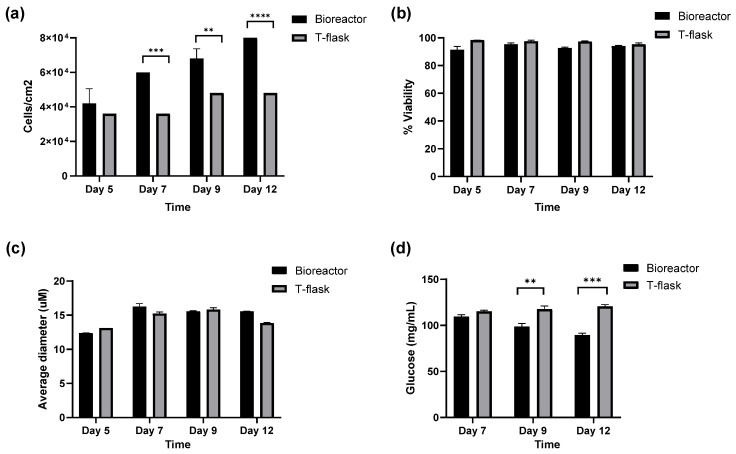
hTERT MSC growth kinetics in a fixed-bed bioreactor versus 2D culture flasks. Cells were harvested from individual sampling strips from the bioreactor and control flasks. Cell counts were performed in parallel to obtain (**a**) cell yield (cells/cm^2^), (**b**) percent viability, and (**c**) average cell diameter. Each cell count was performed in duplicate. (**d**) Media was sampled at each time point to determine the average level of glucose. Each glucose reading was performed in duplicate. ** = *p* < 0.01; *** = *p* < 0.001; **** = *p* < 0.0001. Comparisons without significance markers did not reach statistical significance.

To evaluate the overall performance of the bioreactor for hTERT MSC expansion, we compared cell yield, viability, and glucose consumption against the control flasks across different time points. As shown in Figure 2a, hTERT MSCs grown in the bioreactor displayed a markedly higher total cell yield (viable cells per cm^2^) than control cells grown in standard T-flasks. Notably, cell yield from the bioreactor increased incrementally and consistently at each time point, whereas yield from control flasks was less pronounced and appeared to plateau over time. The increased total cell yield achieved in the bioreactor is attributable to the substantially larger available surface area of the fixed-bed system, which permits higher overall cell numbers to be generated during the final expansion phase without prolonged culture duration or additional serial passaging. Cell viability remained consistently high, with average measurements of greater than 90% across all time points in both platforms (Figure 2b). Additionally, the average cell diameter did not differ significantly between cells cultured in the bioreactor and those grown in control flasks, indicating that the bioreactor environment did not induce hypertrophic changes or alter the physical dimensions of the cultures (Figure 2c). Lastly, glucose consumption over time was significantly higher in the bioreactor culture as compared to the control flasks. The overall glucose levels in the bioreactor decreased over time, whereas the glucose levels in the control flasks remained relatively consistent (Figure 2d). This elevated metabolic activity is consistent with increased cell growth/yield in the bioreactor and suggests a greater demand for energy substrates to support growth. Collectively, these results highlight the potential of the fixed-bed bioreactor as a scalable and efficient platform for the expansion of hTERT MSCs, yielding improved cell growth without compromising cell morphology.

### 3.2. Fixed-Bed Bioreactor Culture Increases EV Yield While Preserving Physical Characteristics

For all EV characterization assays, we compared hTERT MSC EVs isolated from the bioreactor to hTERT MSC EVs that were previously isolated from multi-layer flasks (approximate total surface area ~30,000 cm^2^; approximate total volume ~5 L), which is comparable to the experimental design that was used with the bioreactor system. We rationalized that using EVs isolated from multi-layer flasks as a benchmark would allow us to evaluate whether the bioreactor platform maintains comparable EV quality and characteristics. Furthermore, since our downstream EV isolation methods are standardized, this ensures that any observed differences between bioreactor- and flask-derived EVs can be attributed to the upstream culture platform rather than to variability in isolation techniques.

**Figure 3:**
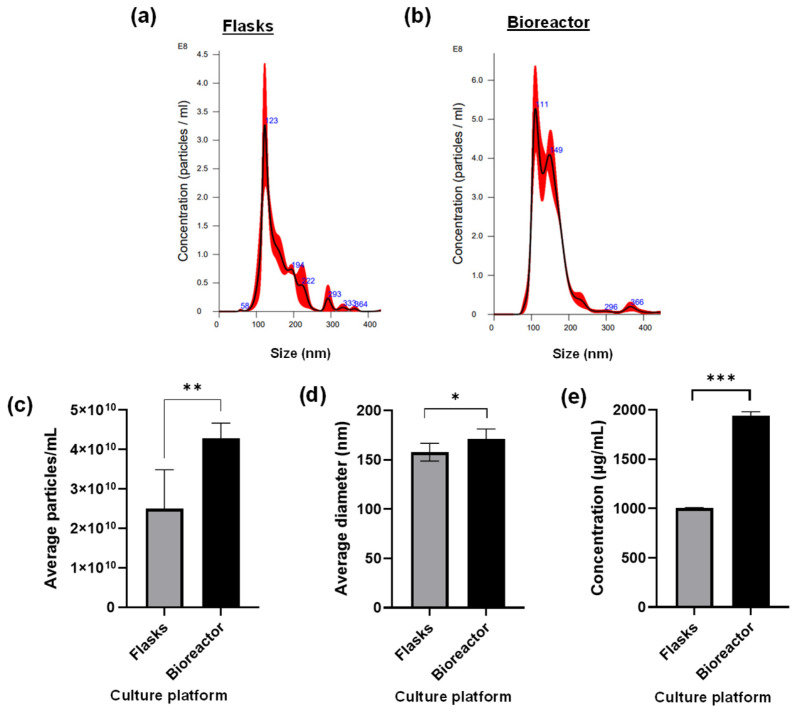
Characterization of EVs produced from a fixed-bed bioreactor vs. 2D culture flasks. NTA was performed on hTERT MSC EVs isolated from (**a**) multi-layer flasks and (**b**) fixed-bed bioreactor to assess the relative concentration and size distribution. Red curves represent the overlaid particle size distribution traces derived from replicate readings of each sample. (**c**) The average particles/mL and (**d**) the average diameter between EV samples are shown. Each sample was analyzed in duplicate with three readings per replicate (n = 6 readings per sample). (**e**) BCA assay was performed to evaluate the average protein concentration of each EV sample. * = *p* < 0.05; ** = *p* < 0.01; *** = *p* < 0.001.

NTA was first performed to measure EV concentration and size. The NTA histograms in Figure 3 show that hTERT MSC EVs from multi-layer flasks have a prominent peak corresponding to 123 nm (Figure 3a), whereas hTERT MSC EVs from the bioreactor have prominent peaks corresponding to 111 nm and 149 nm (Figure 3b). Both histograms also show that the majority of EVs isolated from both platforms are under 200 nm in diameter. The average concentration of EVs isolated from the bioreactor was significantly higher than the concentration of EVs isolated from multi-layer flasks, with values of 4.2 × 10^10^ particles/mL and 2.5 × 10^10^ particles/mL, respectively (Figure 3c). NTA also revealed that EVs isolated from the bioreactor exhibited an average diameter of 171 nm while EVs isolated from multi-layer flasks had an average diameter of 158 nm (Figure 3d). Lastly, to further assess EV quality, we performed protein quantification using BCA assay, which showed that EVs produced in the bioreactor had a significantly higher protein yield than EVs produced in flasks (Figure 3e).

To evaluate the potential impact of freeze–thaw on EV yield, we performed NTA and BCA assays on hTERT MSC EVs before and after a single freeze–thaw cycle. NTA analysis revealed no significant differences in the average particle concentration among pre-freeze and post-freeze samples, indicating that EV recovery was not compromised by the freeze–thaw process (Figure S2a). The overall size distribution remained consistent across samples, with most particles measuring under 200 nm in diameter, suggesting preservation of vesicle morphology (Figure S2b). The average protein concentration was also comparable between samples, further supporting minimal protein degradation or loss during freezing (Figure S2c).

Together, these data confirm that the bioreactor system supports robust EV production while preserving key physical characteristics. The relative concentration, size distribution, and protein yield between platforms indicate that the fixed-bed bioreactor system is suitable for generating EVs at-scale without compromising vesicle integrity. These findings support the integration of bioreactor-based workflows into EV manufacturing where scalability and reproducibility are critical.

### 3.3. Bioreactor-Derived EVs Display Differential Enrichment of Surface Markers

The surface molecules associated with EVs not only define their biological identity but also provide important insights into their potential functional roles in intercellular communication. Therefore, characterizing these markers is essential for validating EV identity and assessing consistency across EV production platforms. To this end, surface marker profiling of hTERT MSC EVs was performed using a multiplex immunoassay platform (MSD). This enabled quantification of sub-populations of EVs expressing each of the canonical tetraspanins and other EV-associated surface molecules, and comparison between the bioreactor and 2D cultures.

**Figure 4:**
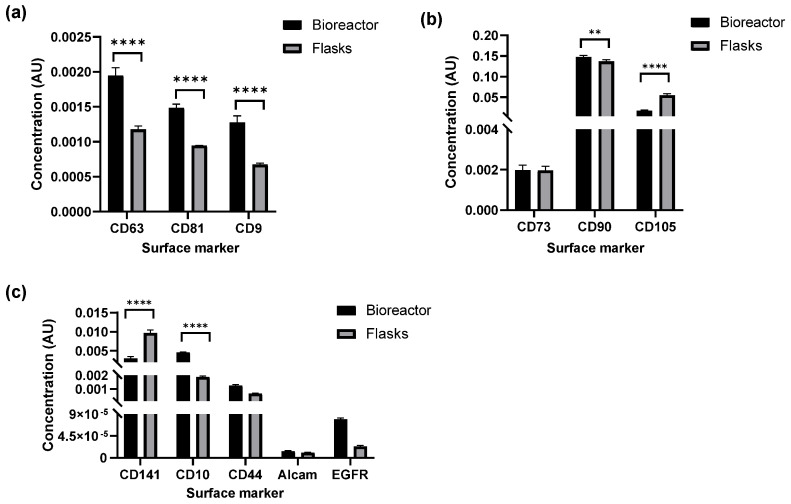
EV surface marker screening. Multiplex analysis was used to assess the expression of EVs with various surface proteins in bioreactor and flask-derived EVs. Each assay was performed in triplicate. Raw ECL values were fit to a standard curve generated using recombinant EV calibrators expressing each surface marker of interest to back-calculate EV concentrations, reported as AU. The relative concentration of EVs with (**a**) EV-associated tetraspanins (CD63, CD81, CD9), (**b**) MSC-associated markers (CD73, CD90, CD105), and (**c**) additional surface markers associated with immune modulation, adhesion, and signaling (CD141, CD10, CD44, Alcam, EGFR) are shown. ** = *p* < 0.01; **** = *p* < 0.0001.

Analysis revealed expression of tetraspanins CD63, CD81, and CD9 in EVs derived from both production methods, confirming their identity. Data in Figure 4a shows the relative concentration of EVs with each of these markers, measured in AU, with significantly higher expression of all three observed in bioreactor-derived EVs. In both EV preparations, the concentration of CD63^+^ EVs was highest, followed by CD81 and CD9. This pattern of relative expression is consistent with our previous findings [22]. To further confirm these findings, we performed Western blot on hTERT MSC EVs produced from the bioreactor. As shown in Figure S3, EVs expressed all three tetraspanins, with CD63 expression being the highest. Additionally, we detected the expression of key EV biogenesis makers, including Alix and Flotillin-1, further supporting their classification as vesicles of endosomal-origin.

Beyond tetraspanin and EV biogenesis marker analysis, EVs were profiled via multiplex immunoassays for MSC-associated surface markers CD73, CD90, and CD105 to further assess phenotypic consistency across production platforms. These markers are commonly used to confirm the cellular origin of EVs and to infer potential immunomodulatory functions. The relative concentrations of these EV populations are shown in Figure 4b. CD73^+^ EVs were detectable at low and comparable levels across both platforms, suggesting minimal enrichment of this marker in EVs. CD90^+^ EVs were significantly higher in bioreactor cultures relative to flasks, suggesting enhanced incorporation under dynamic culture conditions. In contrast, CD105^+^ EVs were more abundant in flask-derived EVs, indicating that static culture may favor the retention or packaging of this marker. These differential expression patterns may reflect subtle shifts in EV biogenesis or cargo selection influenced by the production environment.

To further explore EV surface marker diversity, EVs were evaluated for markers CD141, CD10, CD44, Alcam, and Epidermal Growth Factor Receptor (EGFR). These markers are associated with immune modulation, cell adhesion, and cellular signaling, and their presence on the EV surface may reflect specialized roles in intercellular communication. While all five populations were detected in EVs from both production platforms (Figure 4c), expression patterns varied. CD10 and CD141, both linked to reparative and immunomodulatory functions, were the most abundant among the panel, with CD10 more abundant in bioreactor-derived EVs and CD141 enriched in flask-derived EVs. In contrast, CD44, ALCAM, and EGFR, markers more commonly associated with oncogenic signaling and tumor-derived EVs, were overall less detectable and showed no significant differences between production platforms. These data suggest that EVs produced under both conditions display markers with potential regenerative relevance, while minimizing expression of molecules typically associated with pathological signaling.

### 3.4. Bioreactor Culture Broadens the EV Proteome and Alters Functional Pathway Profiles

**Figure 5:**
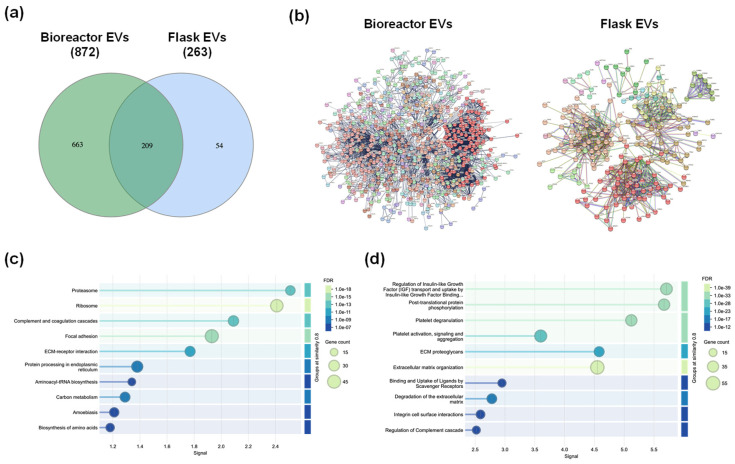
Proteomic analysis of hTERT MSC EVs and pathway enrichment. (**a**) Venn diagram showing the distribution of identified proteins between bioreactor-derived EVs (green) and flask-derived EVs (blue). Bioreactor EVs contained 872 proteins (663 unique) and flask EVs contained 263 proteins (54 unique), with 209 proteins shared. (**b**) STRING network analysis of EV proteins from bioreactor (left) and flask (right) cultures. Protein interaction networks were generated using STRING database with a confidence threshold of 0.7 (high confidence); disconnected nodes were removed. MCA clustering was applied with an inflation parameter of 2 to identify functional clusters. Node colors indicate clusters identified by STRING analysis, as shown by the color key beneath each network. (**c**,**d**) Reactome pathway enrichment bubble plots for (**c**) bioreactor-derived EVs and (**d**) flask-derived EVs. Bubble size indicates the number of mapped protein entries per pathway (reported as gene count by Reactome), and color gradient reflects FDR-adjusted significance.

To further characterize the molecular cargo of hTERT MSC EVs and identify pathways that may contribute to their reparative properties, we performed proteomic profiling using LC-MS/MS. EVs produced from both bioreactor and flask-based cultures were analyzed and protein identification was based on stringent criteria for peptide confidence and FDR. As shown in Figure 5a, bioreactor-derived EVs contained a substantially greater number of proteins (872 total, 663 unique) than flask-derived EVs (263 total, 54 unique), with 209 proteins shared between both populations.

Overall, bioreactor-derived EVs were enriched with proteins related to translation and ribosome machinery (e.g., EIF3A, EIF4E, EIF6, ETF1, various RPL proteins), proteasome regulation (e.g., PSMD5, PSME2, PSME1, PSMA7, PSMB4, PSMB5, PSMD2, PSMD1), extracellular matrix (ECM) remodeling (e.g., COL4A5, LOX3), and signaling receptors (e.g., PDGFRA, PDGFRB). Flask-derived EVs were enriched with proteins related to coagulation and complement regulation (e.g., ADAMTS13, FGB, FGG, CFH), antioxidant defense (e.g., GPX3, SOD3), lysosomal function (e.g., CLN5), and RNA polymerase activity (e.g., TAF7). Proteins common to both EV platforms were mainly associated with ECM organization, cell adhesion, and cytoskeletal structure (e.g., FN1, VIM, THBS1, COL1A1, COL1A2).

STRING analysis followed by MCA clustering revealed platform-specific differences in cargo composition. As shown in Figure 5b, bioreactor-derived EVs formed interconnected networks with multiple dense clusters that were dominated by proteins involved in cytoplasmic translation, ECM organization, protein folding, and ER-to-Golgi vesicle-mediated transport. In contrast, flask-derived EVs displayed fewer and less interconnected clusters that were primarily associated with ECM proteoglycans, blood microparticle components, proteasome subunits, and antioxidant activity. Reactome pathway enrichment (Figure 5c,d) further highlighted these differences. For example, bioreactor-derived EVs were enriched for pathways related to translation initiation and elongation, protein folding, and signaling by Roundabout (ROBO) receptors, as well as regulation of Slit ligand (SLIT)/ROBO expression and IGF signaling. These pathways suggest a cargo profile that supports protein synthesis and intracellular trafficking, potentially contributing to enhanced reparative capacity. In contrast, flask-derived EVs were enriched for pathways associated with post-translational protein phosphorylation, platelet activation and degranulation, ECM proteoglycans, ECM organization, and ECM degradation. This enrichment pattern indicates a functional bias toward ECM remodeling and platelet-related processes, which may influence hemostasis and tissue repair in a manner distinct from the protein synthesis and signaling functions observed in bioreactor-derived EVs. Additional functional enrichment analyses, including GO and KEGG pathway results, are provided in Figure S4. Together, these findings demonstrate that bioreactor culture conditions not only increase EV yield but also shape molecular composition in ways that may enhance reparative properties.

### 3.5. Bioreactor-Derived EVs Exhibit Enhanced Reparative Activity In Vitro

Given the proteomic enrichment of proteins associated with translation, ECM remodeling, and cell cycle regulation, we next sought to determine whether hTERT MSC EVs produced in the fixed-bed bioreactor retained function activity. To assess this, we performed a set of in vitro assays focused on cellular responses to genotoxic stress. Given the therapeutic relevance of skin regeneration and repair, human keratinocytes were selected to evaluate how EV-mediated reparative activities affect radiation-induced damage. These studies build on our previous findings demonstrating the in vitro reparative effects of stem cell EVs in ocular and CNS cells and extend this work to skin cells. This experimental approach allowed us to evaluate the ability of EVs to influence cell repair through both phenotypic and molecular readouts.

**Figure 6:**
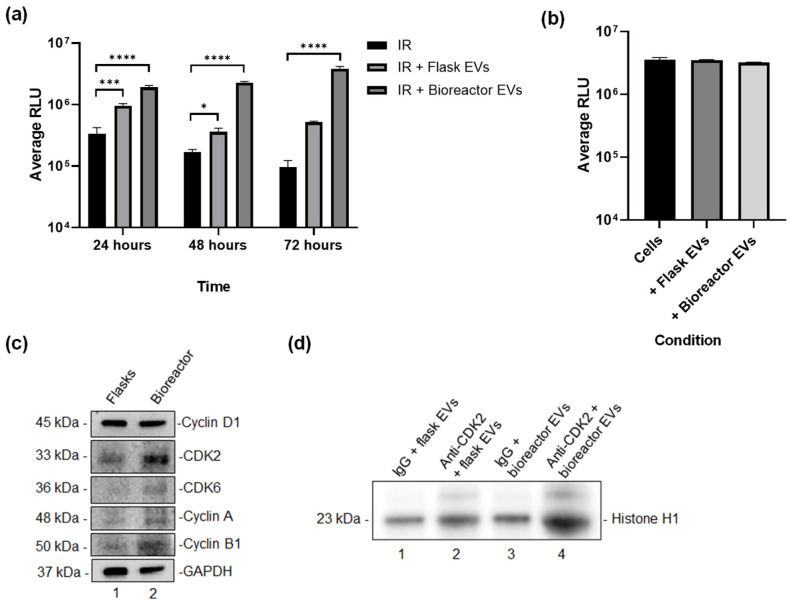
EV function and signaling activity. (**a**) Primary keratinocytes were exposed to IR and treated with EVs from either flask or bioreactor cultures. Cell viability was assessed at 24, 48, and 72 h using the CellTiter-Glo assay. (**b**) Non-irradiated keratinocytes were treated with EVs from flask or bioreactor cultures, and viability was assessed after 72 h using the same assay. * = *p* < 0.05; *** = *p* < 0.001; **** = *p* < 0.0001. (**c**) Western blot of cell cycle proteins associated with hTERT MSC EVs produced from both the bioreactor and flask-based cultures. (**d**) In vitro kinase assay of EV-associated CDK2. EVs were immunoprecipitated with CDK2 antibodies or IgG control and kinase activity was assessed using Histone H1 as substrate.

To assess the reparative potential of EVs, we first examined their ability to promote recovery following radiation-induced damage, a model relevant to skin injury and repair. In this assay, primary human epidermal keratinocytes were exposed to ionizing radiation and subsequently treated with EVs produced from either the bioreactor or flask-based cultures. Cell viability was measured over a 72-h time course using the CellTiter-Glo assay. As shown in Figure 6a, both EV preparations improved cell viability relative to irradiated controls; however, bioreactor-derived EVs consistently demonstrated a stronger effect at every time point (24, 48, and 72 h). At 24 h, bioreactor EVs produced a rapid and pronounced increase in viability compared to flask-derived EVs, suggesting an early protective response. This advantage persisted at 48 h, where bioreactor EVs continued to outperform flask EVs, indicating sustained reparative activity during the recovery phase. By 72 h, bioreactor EV-treated cells achieved the highest viability overall, highlighting their potential to support long-term restoration of cellular health. To confirm that EV treatment does not alter viability in undamaged cells, we performed a parallel assay using non-irradiated keratinocytes. As shown in Figure 6b, baseline viability remained high and comparable across all conditions, indicating that EVs do not adversely impact viability under normal conditions.

To further confirm functional activity across different skin cell types, we performed cell migration assays using hTERT-immortalized keratinocytes and human dermal fibroblasts treated with EVs from either hTERT MSCs (bioreactor-derived) or PC3 (prostate cancer) cells. As shown in Figure S5, hTERT MSC EVs enhanced migration in both dermal fibroblasts (Figure S5a) and keratinocytes (Figure S5b) as compared to untreated controls, whereas PC3 EVs exhibited only partial closure over a three-day incubation period. Quantitative analysis revealed distinct migration dynamics between the two cell types. Dermal fibroblasts (Figure S5c) responded more gradually to hTERT MSC EV treatment, achieving near-complete gap closure by Day 3, whereas keratinocytes (Figure S5d) exhibited a rapid response, reaching complete closure within 24 h. Treatment with PC3 EVs improved migration in both models but to a lesser extent than hTERT MSC EVs.

Building on these functional observations, we next sought to determine whether differences in molecular cargo could potentially explain the enhanced reparative properties observed with bioreactor-derived EVs. Because tissue repair requires tightly coordinated progression through the cell cycle, we focused on proteins that regulate cell cycle transitions and support controlled proliferation during recovery. Western blot revealed that EVs produced under bioreactor conditions had higher expression of several key regulators, including Cyclin D1, Cyclin A, Cyclin B1, CDK2, and CDK6 (Figure 6c). These findings suggest that the dynamic conditions of the bioreactor may enhance packaging of cell cycle-associated proteins into EVs.

Finally, to determine whether these proteins may retain functional activity, we performed an in vitro kinase assay using Histone H1 as a substrate. EV lysates were immunoprecipitated with anti-CDK2 antibodies, and phosphorylation of Histone H1 was assessed as a readout of CDK2 activity. EV lysates from both bioreactor and flask cultures were immunoprecipitated using anti-CDK2 antibodies, with IgG used as a negative control. Kinase activity was assessed by measuring the phosphorylation of Histone H1, a well-established CDK2 target. As shown in Figure 6d, bioreactor-derived EVs exhibited higher kinase activity compared to flask-derived EVs, confirming that cell cycle proteins within these vesicles are enzymatically active and may contribute to enhanced reparative properties. Collectively, these data demonstrate that hTERT MSC EVs produced under dynamic bioreactor conditions not only maintain functional activity but also exhibit elevated molecular signaling capacity relative to flask-derived EVs.

## 4. Discussion

With the rapid growth of EV research and the accelerating interest in EV-based applications, developing scalable, standardized production platforms has become a critical priority. Consequently, there is a demand for robust, high-capacity manufacturing platforms that ensure consistency without compromising quality or biological activity. Current methods based on planar, multi-layer flasks are relatively labor-intensive and offer limited control over process parameters. These constraints not only limit production capacity but also have the potential to introduce variability that could compromise reproducibility and downstream consistency. Automated systems, including bioreactors, that maintain environmental control and minimize operator-dependent variability are a promising solution for overcoming current challenges and enabling consistent, high-yield EV manufacturing.

In addition to platform scalability, the cellular source is equally important for ensuring consistency and reproducibility. To support large-scale production while maintaining functional integrity, the producing cells must deliver high yields without compromising biological quality. MSC-derived EVs are widely regarded for their reparative properties, yet primary MSCs pose certain challenges such as donor variability and limited proliferative capacity. MSCs that have been immortalized via hTERT expression overcome these challenges by providing a stable, well-characterized source that enables extended culture without senescence-related issues. In our previous work, we demonstrated that hTERT MSCs can produce EVs that retain the key physical, biochemical, and functional properties associated with primary MSCs [22]. This study provides proof-of-concept evidence that a fixed-bed bioreactor platform can be leveraged to produce EVs from hTERT MSCs while maintaining, or even enhancing, key physical, biochemical, and functional attributes relative to matched 2D flask-based production.

Through direct comparison of EVs produced from the same cell type under equivalent downstream processing conditions, we established that differences in yield, cargo, and activity likely stem from the upstream culture environment. By coupling continuous control of pH, oxygen, and temperature with media recirculation, the bioreactor maintains a balanced and homogeneous culture environment that promotes healthy cell growth at high density. Importantly, the system provides a low-shear environment, creating gentle culture conditions for adherent-based cells. ICC staining of hTERT MSCs (Figure S6) further illustrates their distribution throughout the fibrous matrix, consistent with a 3D pattern rather than a planar monolayer. This spatial arrangement, combined with reduced shear, may be particularly advantageous for EV production, as elevated shear stress has been shown to trigger cell cycle arrest in various cell types [38,39,40]. Compared to other bioreactor systems, such as stirred tank bioreactors that often expose cells to higher shear forces [41], the fixed-bed configuration may therefore be ideal for maximizing EV yield and preserving vesicle identity. While our data confirms enhanced EV output, the precise cell-cycle distribution under these conditions remains unknown. It is possible that dynamic culture favors phases such as late S or G2/M, where translation and vesicle biogenesis are most active. Future studies will incorporate cell-cycle staining to map these kinetics and determine whether phase enrichment correlates with EV size and cargo composition.

In addition to demonstrating scalability, it was also important to verify that bioreactor-derived EVs retained defining molecular features. Surface marker profiling confirmed the presence of EVs with canonical tetraspanins (CD63, CD81, CD9) in both production platforms, demonstrating vesicle identity. The pattern of expression (CD63 > CD81 > CD9) was consistent across both production platforms and aligns with our previous observations, highlighting the reproducibility of EV phenotypes under different culture conditions. Interestingly, bioreactor-derived EVs exhibited higher levels of these markers despite being normalized for particle input, possibly indicating greater marker density per vesicle. This enrichment may reflect differences in vesicle composition or shifts in EV biogenesis pathways under the dynamic culture conditions of the bioreactor. EVs were also screened for MSC-associated markers and other signaling proteins, revealing differences in expression of subpopulations with potential functional significance. For example, bioreactor cultures exhibited enrichment of EVs with CD90 (Thy-1) and CD10 (Neprilysin), markers associated with immunomodulatory and reparative functions [42,43,44,45], whereas EVs with CD105 (Endoglin) and CD141 (Thrombomodulin), linked to angiogenesis and vascular homeostasis [46,47,48,49], were relatively reduced compared to flask-based cultures. These compositional shifts suggest that dynamic culture may favor EV phenotypes that are optimal for immune regulation and tissue repair. Notably, EVs with markers commonly associated with oncogenic signaling (e.g., EGFR and ALCAM) were detected at concentrations several orders of magnitude lower than the MSC specific surface proteins, with no significant difference between the culture platforms. This profile further supports the regenerative potential of these vesicles while minimizing concerns about pro-tumorigenic signaling.

Building on these findings, proteomic analysis revealed distinct differences in EV cargo, with bioreactor-derived EVs displaying a markedly broader and more complex proteome relative to flask-derived vesicles. This increase in protein diversity suggests that the dynamic culture conditions of the bioreactor not only increase EV yield but also expand their molecular complexity, potentially enhancing their functional capacity. The dense networks observed in bioreactor-derived EVs were dominated by clusters linked to cytoplasmic translation and protein folding, indicating a cargo profile that could influence protein homeostasis and deliver translation-related proteins capable of modulating mRNA processing and synthesis in recipient cells. These features, combined with enrichment for ECM remodeling proteins and signaling receptors such as PDGFRA/B, indicate a molecular composition that supports tissue remodeling and cellular recovery. In contrast, flask-derived EVs exhibited a narrower proteomic signature with fewer and less interconnected clusters. Their cargo was also enriched for components central to tissue repair, including ECM organization, platelet activation, and antioxidant activity, all of which are involved in wound stabilization and early regenerative processes.

Because bioreactor-derived EVs contained a greater number of detectable proteins, we also evaluated whether this increase might reflect non-EV-associated protein carryover rather than true compositional differences. However, both platforms used consistent TFF parameters, minimizing the potential for variations to be introduced during downstream processing. In-process controls, including viability, morphology, and glucose consumption, likewise did not indicate increased cell damage or release of intracellular proteins under bioreactor conditions. Importantly, the enriched proteins formed coherent functional clusters rather than the diffuse background expected from non-vesicular protein carryover. Taken together with the significantly higher EV yield, these observations suggest that dynamic culture conditions enhance the incorporation of EV-associated proteins rather than increasing non-vesicular protein background.

A particularly striking finding was the enrichment of SLIT/ROBO signaling components in bioreactor-derived EVs. SLIT ligands and ROBO receptors are well known for their roles in neuronal development and axon guidance. However, these molecules are also increasingly recognized as regulators of tissue architecture beyond the nervous system, influencing cell migration, angiogenesis, and epithelial integrity, processes that are essential for wound healing and regeneration [50,51,52,53]. Their association with EVs suggests a mechanism by which these vesicles could influence spatial organization and directional migration of cells in damaged tissue, complementing the structural and metabolic support provided by other protein clusters. This signaling axis, together with IGF-related pathways also detected in bioreactor EVs, highlights the potential for these vesicles to actively modulate regenerative signaling networks rather than serving solely as passive carriers of structural proteins. Future studies will further explore whether SLIT/ROBO enrichment in bioreactor-derived EVs translates into functional benefits for tissue repair. This could include in vitro migration and angiogenesis assays performed with or without pathway inhibitors, alongside evaluation of downstream signaling molecule expression. In parallel with proteomic analysis, we also evaluated EV-associated RNA. KEGG pathway grouping revealed high-level representation of core cellular processes across both platforms, including genetic information processing (e.g., transcription, translation, replication, and repair), signal-transduction, and membrane transport pathways (Figure S7a,b). As expected for these broad KEGG super-pathways, none differed significantly between platforms. In contrast, GO enrichment analysis provided finer resolution, revealing platform-linked differences in EV-associated transcripts (Figure S7c,d). Bioreactor-derived EVs demonstrated relatively stronger enrichment of immune receptor–interaction terms (e.g., immunoglobulin receptor binding and CCR chemokine receptor binding) and intermediate filament organization, whereas flask-derived EVs showed more prominent enrichment of ECM-associated categories and immunoglobulin complex/humoral immune response pathways. As these findings were descriptive and not experimentally validated, they are interpreted as preliminary indications that culture conditions can affect multiple dimensions of EV transcript composition. Future experiments will incorporate validations and mechanistic experiments to assess whether the observed cargo differences translate into measurable effects on EV-mediated repair.

Lastly, to determine whether compositional differences may translate into functional relevance, we evaluated the effects of EVs in vitro using a model of genotoxic stress. Both bioreactor- and flask-derived EVs improved keratinocyte recovery following radiation injury, confirming that hTERT MSC EVs retain their well-documented reparative capacity regardless of production platform. However, the accelerated increase in viability observed in cells treated with bioreactor EVs suggests that these vesicles may confer additional benefits beyond baseline repair. Additionally, the accelerated recovery observed with bioreactor EVs may reflect synergy between structural/metabolic support and signaling activation. While proteomic enrichment of translation machinery and SLIT/ROBO components suggests vesicles that reinforce protein homeostasis and guide repair, functional assays indicate earlier and more efficient cell-cycle re-entry. For example, irradiated keratinocytes treated with bioreactor-derived EVs showed increased Cyclin A, Cyclin E, and Cyclin C, consistent with enhanced regulation of the G1/S transition and S-phase progression. This was further supported by parallel kinase assays, which showed increased CDK2 activity in bioreactor-derived EVs, consistent with activation of cyclin–CDK complexes that drive checkpoint recovery and S-phase entry. We therefore suspect that bioreactor-derived EVs are more efficient in accelerating S-phase entry and facilitating DNA repair following genotoxic stress, potentially through enrichment of components that enhance cyclin-CDK signaling and promote cell-cycle progression.

In addition to protein cargo that may support cell-cycle re-entry, it is also possible that non-coding RNAs associated with EVs contribute to the enhanced S-phase progression we observed. Long non-coding RNAs have well-recognized roles in regulating cell-cycle transition and checkpoint recovery. For example, lncRNA MALAT1 has been shown to modulate the G1/S transition and mitotic progression by regulating expression of transcription factors alternative splicing of cell-cycle factors [54], while the MYC-regulated lncRNA CONCR is essential for sister chromatid cohesion via its interaction with the DNA helicase DDX11 [55]. Additionally, the lncRNAs LINC00704, LUCAT1, and MIAT originating from intergenic spacers were identified as being essential for S-phase progression [56]. While a complete characterization of EV-associated lncRNA was beyond the scope of this study, these examples highlight an additional layer of regulatory potential within EV-associated cargo. Subsequent work will assess the levels and potential specificity of lncRNAs from hTERT MSC bioreactor-derived EVs and evaluate their regulatory targets in damaged cells.

Taken together, this work establishes a proof-of-concept framework for scalable EV manufacturing using hTERT MSCs in a fixed-bed bioreactor. By integrating a stable cell line with a controlled, 3D environment, we demonstrate that bioreactor-based production not only improves EV yield but also preserves, and in some cases potentially enhances, the molecular complexity and reparative functionality of these vesicles. The enrichment of translation-related proteins, signaling mediators, and cell-cycle regulators in bioreactor-derived EVs suggests a cargo profile that actively supports tissue recovery and cellular homeostasis. These findings underscore the potential of combining hTERT MSCs with bioreactor technology to overcome current limitations in EV scalability and reproducibility, providing a foundation for more standardized workflows that can meet the demands of preclinical applications. By addressing key challenges in EV manufacturing, this work supports an important requirement for future translational research efforts.

## Data Availability

The original contributions presented in this study are included in the article/Supplementary Material. Further inquiries can be directed to the corresponding author(s).

## Supplementary Materials

The following supporting information can be downloaded at https://www.mdpi.com/article/10.3390/cells15070654/s1, Figure S1: Full-length Western blots; Figure S2: NTA and BCA comparing hTERT MSC EVs pre- and post-freeze; Figure S3: Western blot of hTERT MSC EVs; Figure S4: Cell migration assay; Figure S5: GO and KEGG enrichment for proteomics of hTERT MSC EVs; Figure S6: immunocytochemical images of hTERT MSCs cultured in the scale-X hydro fixed-bed bioreactor; Figure S7: KEGG and GO enrichment for RNA-seq of hTERT MSC EVs.

## Abbreviations

The following abbreviations are used in this manuscript:

2D	Two-Dimensional
3D	Three-Dimensional
AU	Arbitrary Units
ATCC	American Type Culture Collection
BCA	Bicinchoninic Acid Protein Assay
CC	Concentration Control
CPAT	Coding-Potential Assessment Tool
CPC	Coding Potential Calculator
CNCI	Coding-Noncoding Index
ECM	Extracellular Matrix
ECL	Electrochemiluminescence
EGFR	Epidermal Growth Factor Receptor
EV	Extracellular Vesicle
FDR	False Discovery Rate
FPKM	Fragments Per Kilobase of Transcript per Million Mapped Reads
GO	Gene Ontology
hTERT	Human Telomerase Reverse Transcriptase
iPSC	Induced Pluripotent Stem Cell
IR	Ionizing Radiation
KEGG	Kyoto Encyclopedia of Genes and Genomes
LC-MS/MS	Liquid Chromatography–Tandem Mass Spectrometry
MCA	Markov Cluster Algorithm
MSC	Mesenchymal Stem Cell
MWCO	Molecular Weight Cut-Off
NTA	Nanoparticle Tracking Analysis
Pfam	Protein families database
ROBO	Roundabout receptor
SLIT	Slit ligand
STRING	Search Tool for the Retrieval of Interacting Genes/Proteins
TFF	Tangential Flow Filtration
