# Supplementary material for: Fixed-Bed Bioreactor Culture Enhances Yield and Reparative Properties of hTERT Mesenchymal Stem Cell Extracellular Vesicles
Source: Cells. 2026 Apr 7;15(7):654. doi: 10.3390/cells15070654 (PMC13073658; doi:10.3390/cells15070654)
Supplement: Supplementary file 1 [file cells-15-00654-s001.zip › cells-4207804-supplementary.pdf]

(a) Cyclin D1 (b) Cdk2 (c) Cdk6

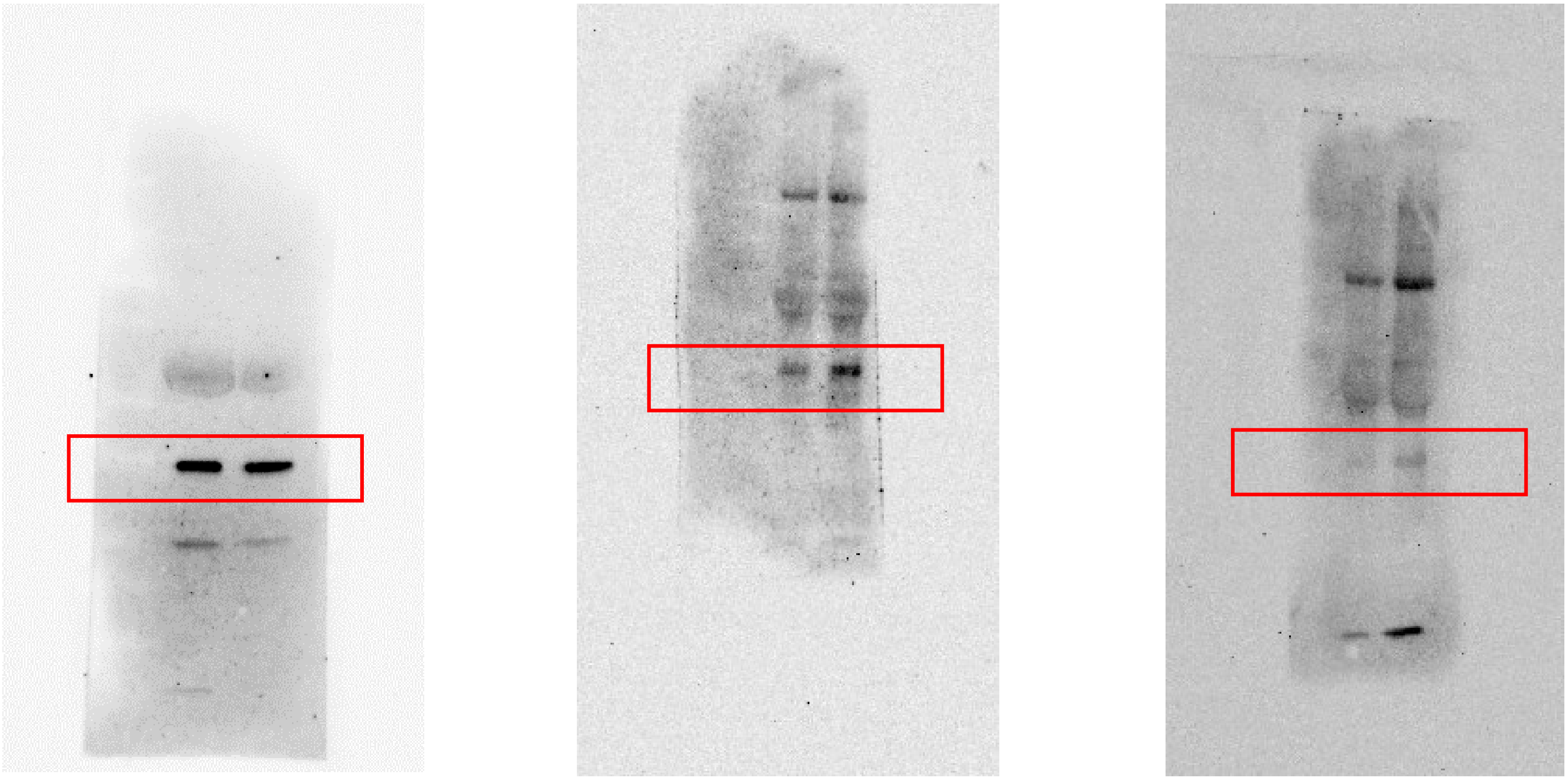

Figure S1

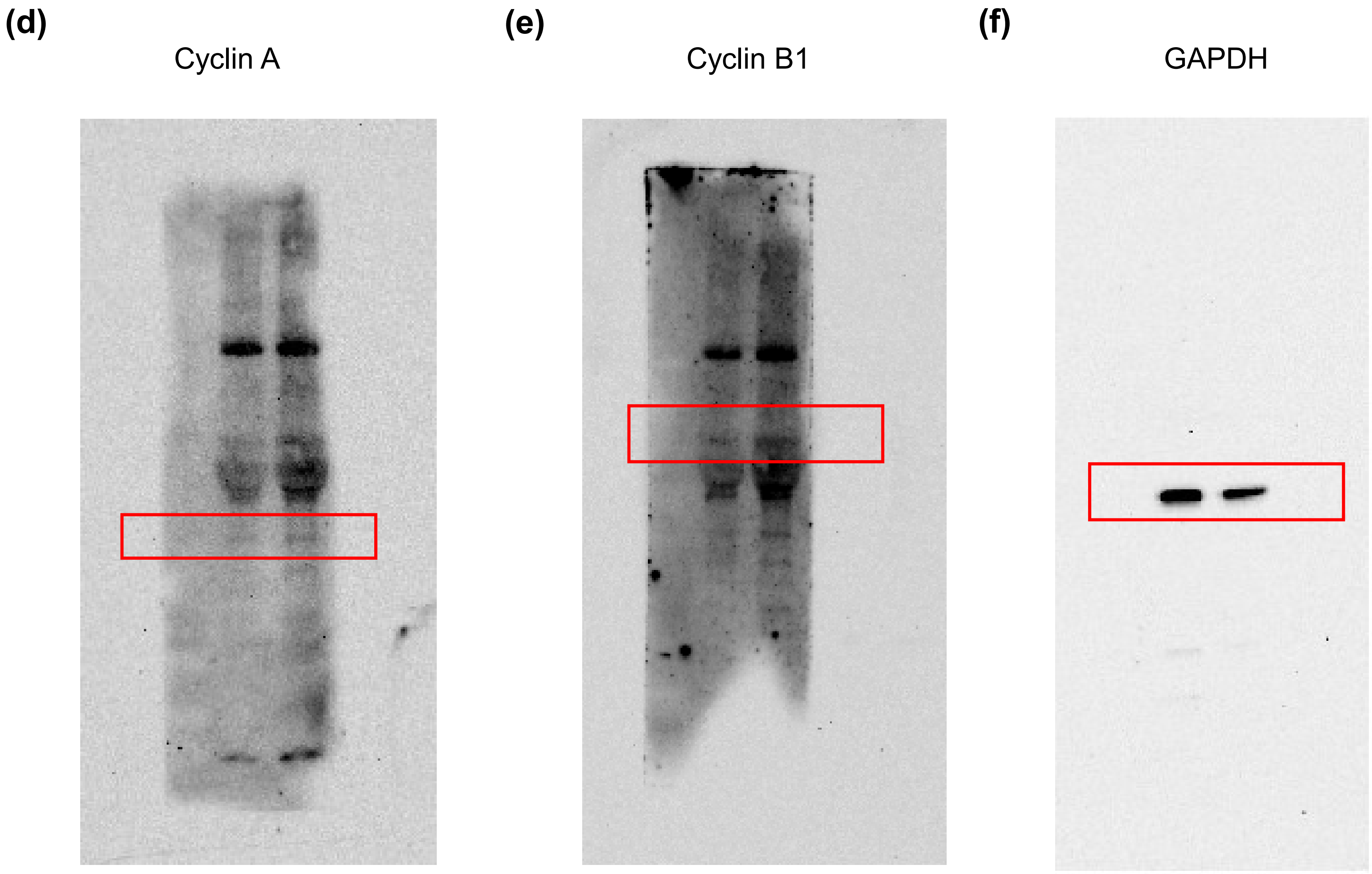

Figure S1

(g)

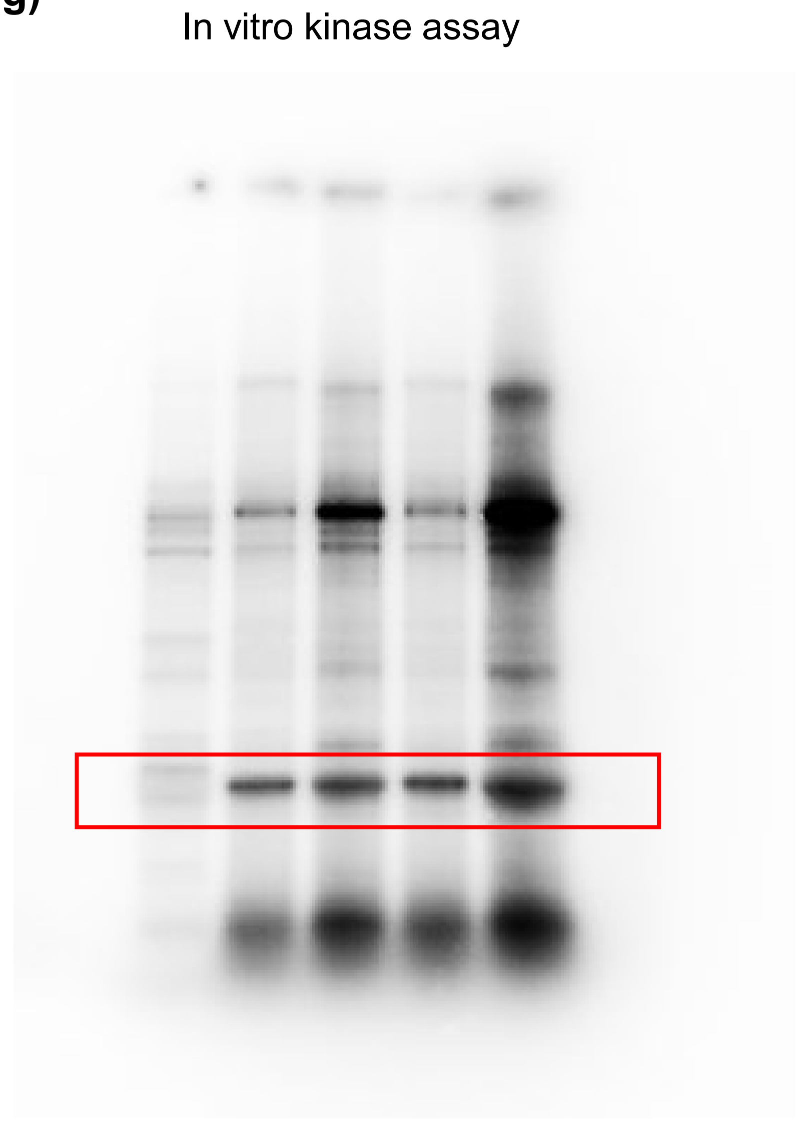

Figure S1

(h)

CD63

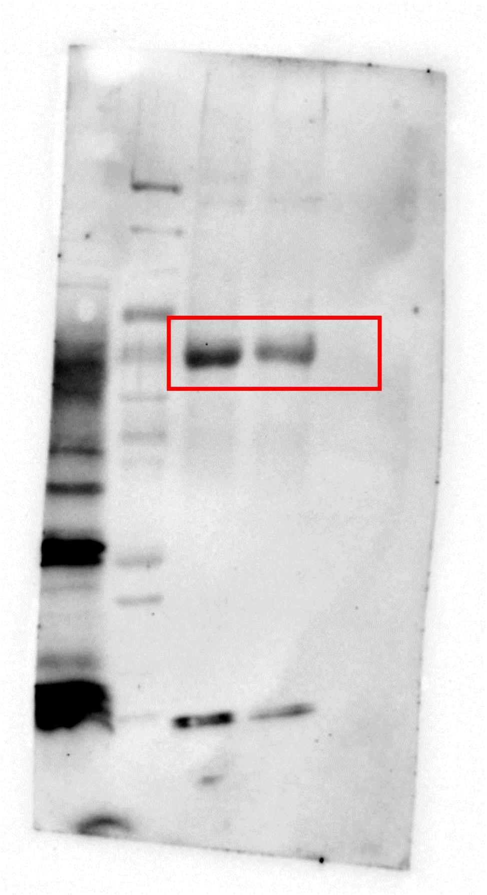

(i)

CD9

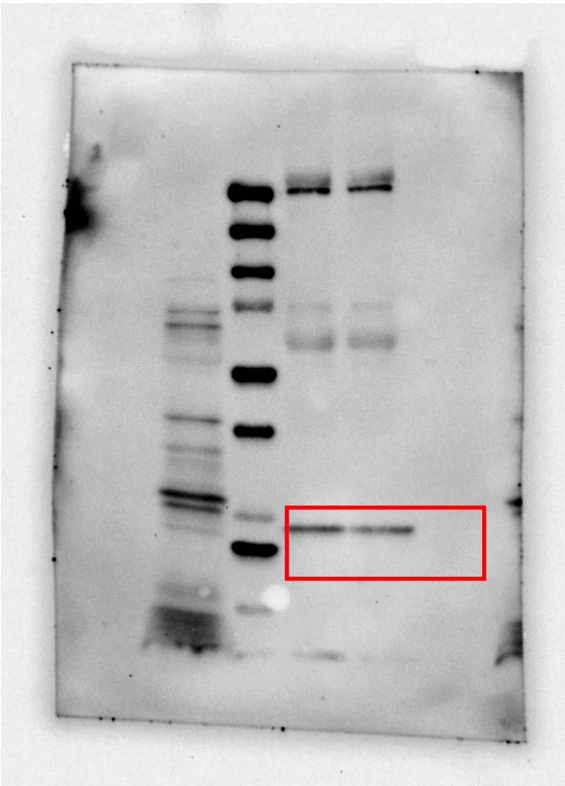

(j)

CD81

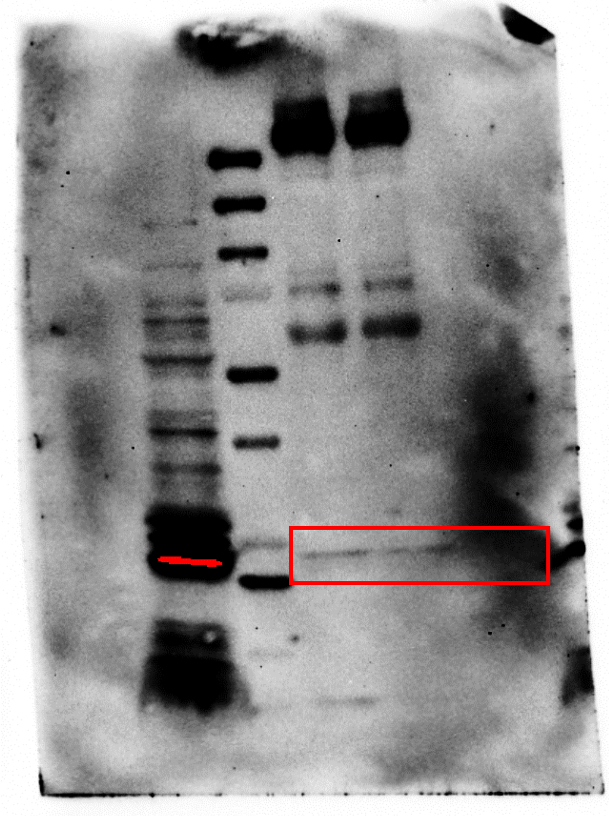

Figure S1

(k) Flotillin-1

(l) Alix

(m) Actin

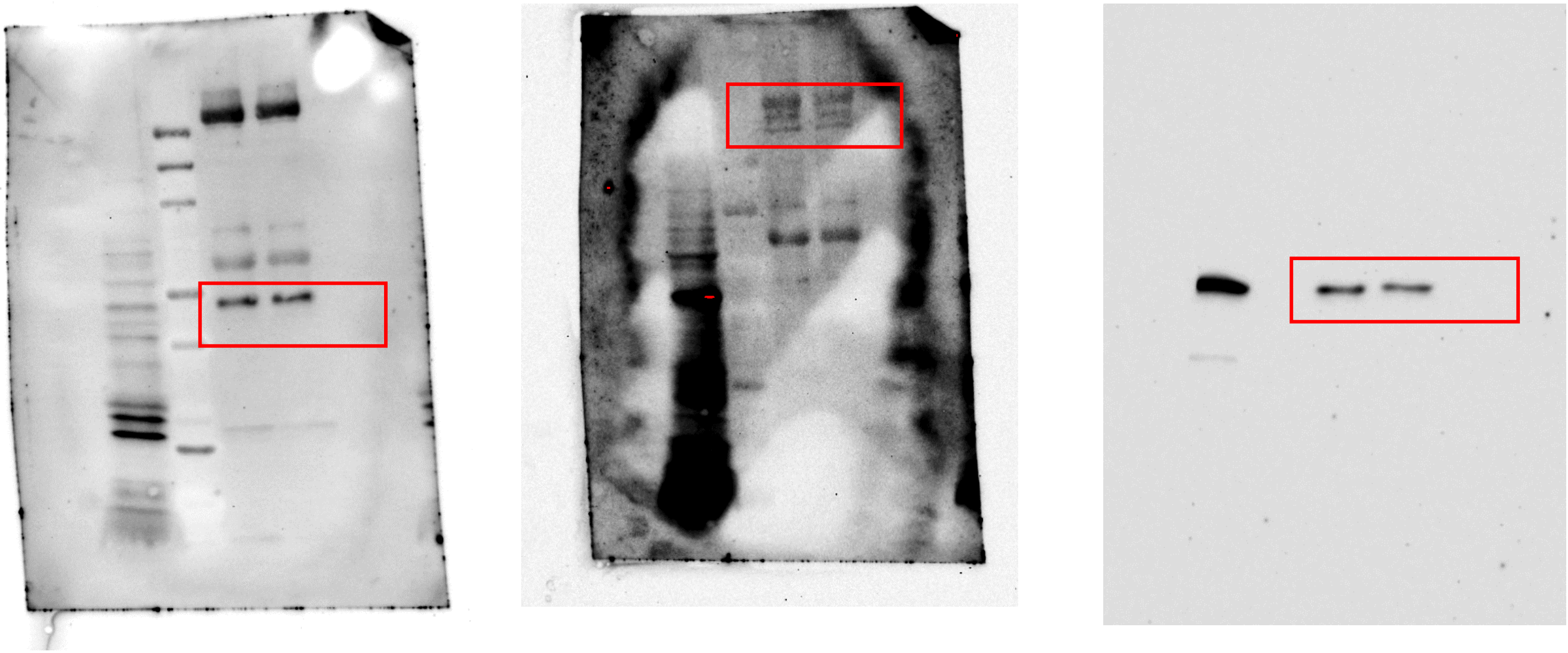

Figure S1

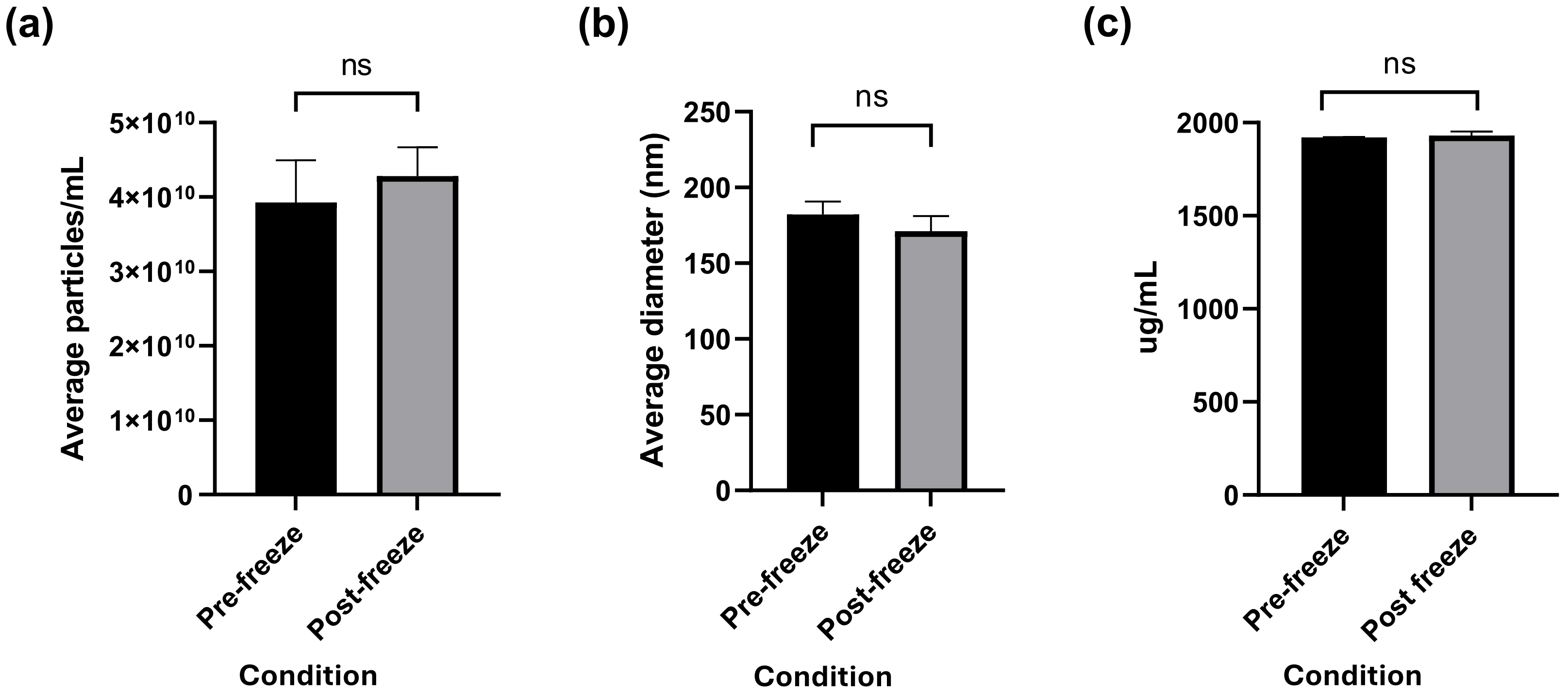

(a)
ns
5×10¹⁰
4×10¹⁰
3×10¹⁰
2×10¹⁰
1×10¹⁰
0
Average particles/mL
Pre-freeze
Post-freeze
Condition
(b)
ns
250
200
150
100
50
0
Average diameter (nm)
Pre-freeze
Post-freeze
Condition
(c)
ns
2000
1500
1000
500
0
ug/mL
Pre-freeze
Post freeze
Condition

Figure S2

(a)

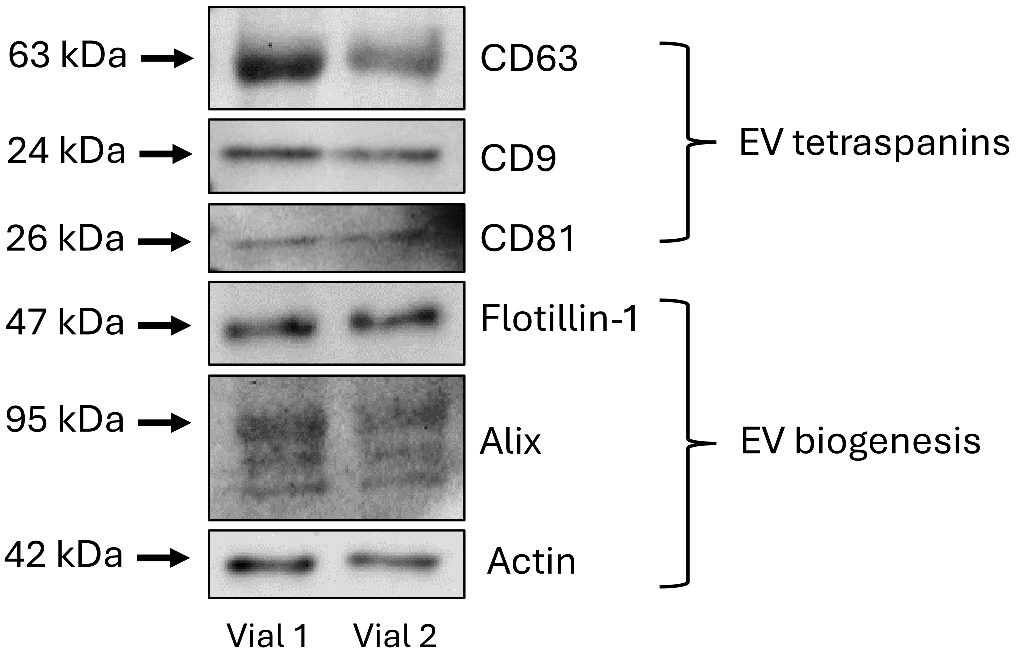
63 kDa
CD63
24 kDa
CD9
26 kDa
CD81
EV tetraspanins
47 kDa
Flotillin-1
95 kDa
Alix
42 kDa
Actin
EV biogenesis
Vial 1
Vial 2

Figure S3

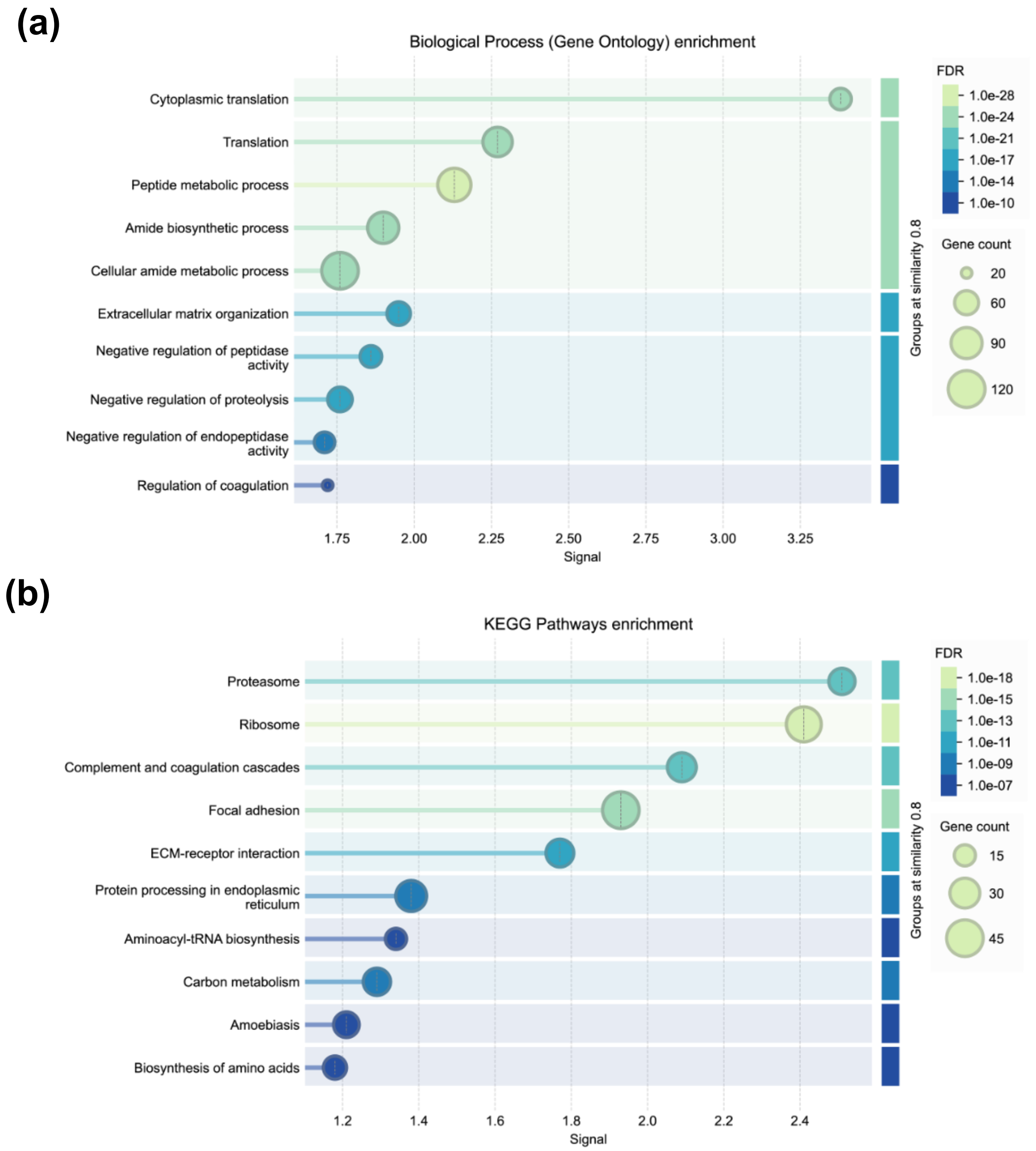
(a)
Biological Process (Gene Ontology) enrichment
Cytoplasmic translation
Translation
Peptide metabolic process
Amide biosynthetic process
Cellular amide metabolic process
Extracellular matrix organization
Negative regulation of peptidase activity
Negative regulation of proteolysis
Negative regulation of endopeptidase activity
Regulation of coagulation
1.75
2.00
2.25
2.50
2.75
3.00
3.25
Signal
Groups at similarity 0.8
FDR
1.0e-28
1.0e-24
1.0e-21
1.0e-17
1.0e-14
1.0e-10
Gene count
20
60
90
120
(b)
KEGG Pathways enrichment
Proteasome
Ribosome
Complement and coagulation cascades
Focal adhesion
ECM-receptor interaction
Protein processing in endoplasmic reticulum
Aminoacyl-tRNA biosynthesis
Carbon metabolism
Amoebiasis
Biosynthesis of amino acids
1.2
1.4
1.6
1.8
2.0
2.2
2.4
Signal
Groups at similarity 0.8
FDR
1.0e-18
1.0e-15
1.0e-13
1.0e-11
1.0e-09
1.0e-07
Gene count
15
30
45

Figure S4

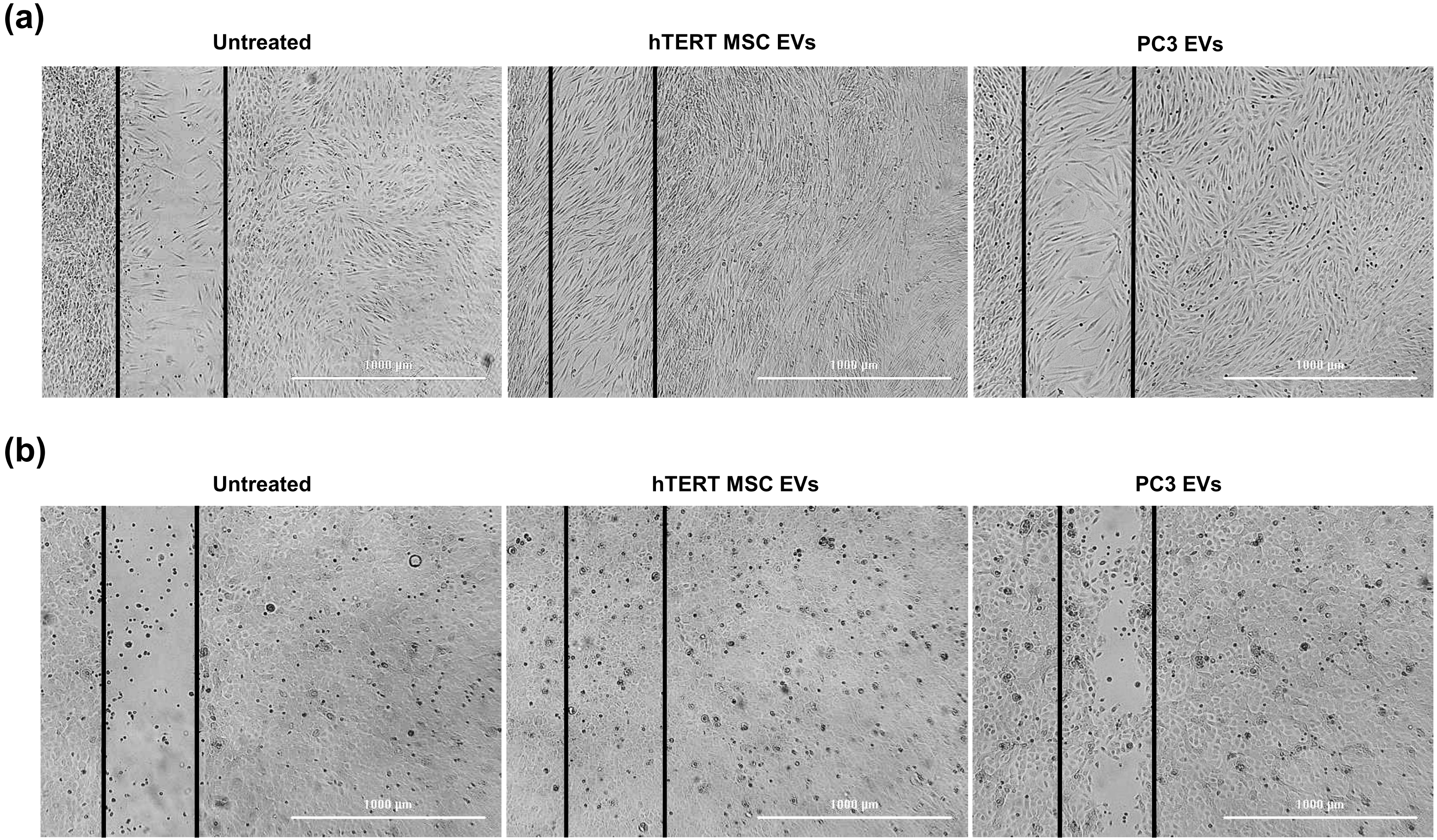
(a)
Untreated
hTERT MSC EVs
PC3 EVs
1000 µm
1000 µm
1000 µm
(b)
Untreated
hTERT MSC EVs
PC3 EVs
1000 µm
1000 µm
1000 µm

Figure S5

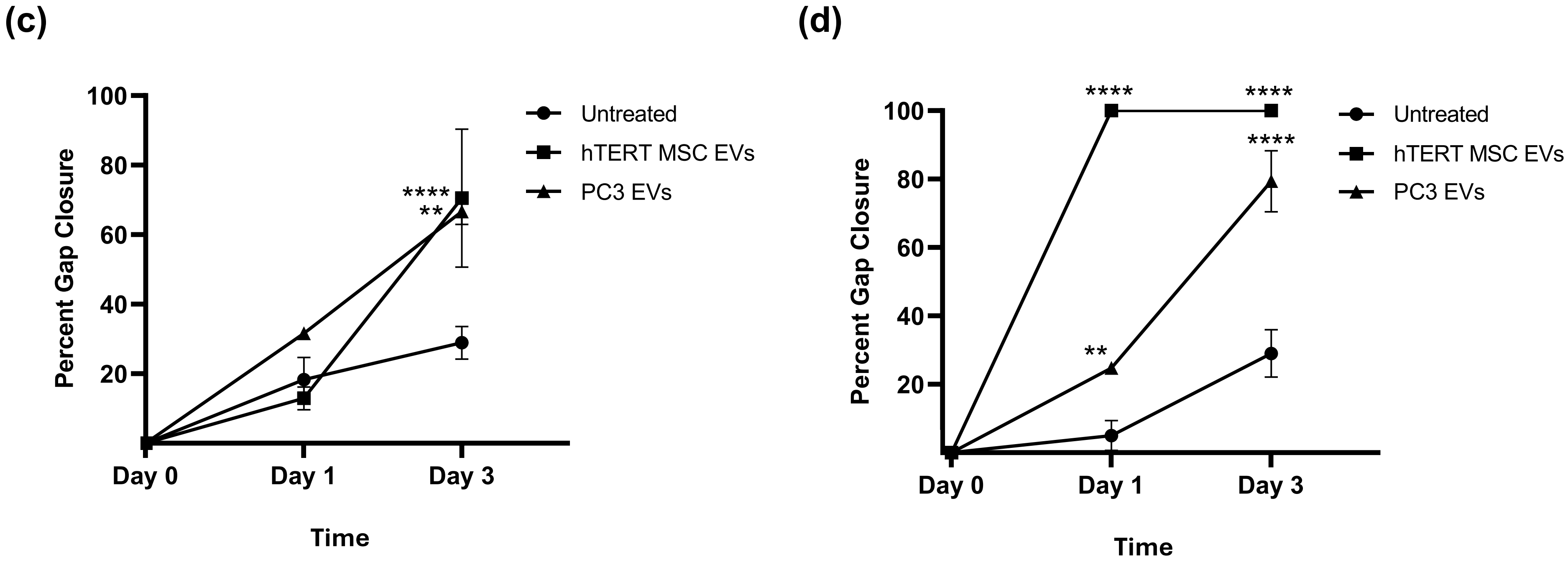

(c)
Percent Gap Closure
100
80
60
40
20
Day 0
Day 1
Day 3
Time
****
**
Untreated
hTERT MSC EVs
PC3 EVs
(d)
Percent Gap Closure
100
80
60
40
20
Day 0
Day 1
Day 3
Time
****
****
****
**
Untreated
hTERT MSC EVs
PC3 EVs

Figure S5

(a)

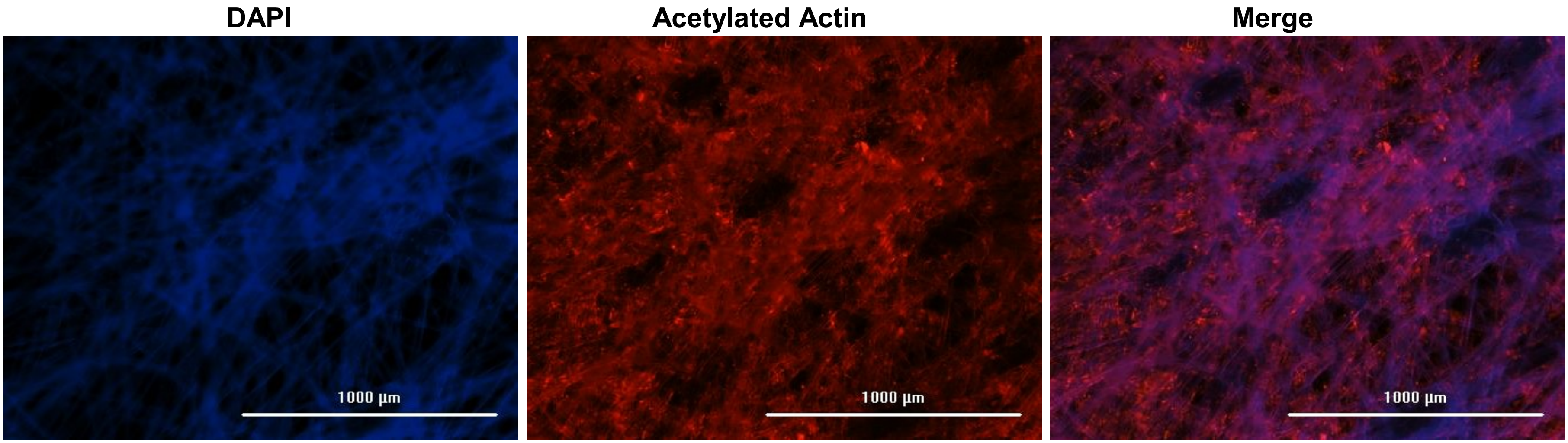

Figure S6

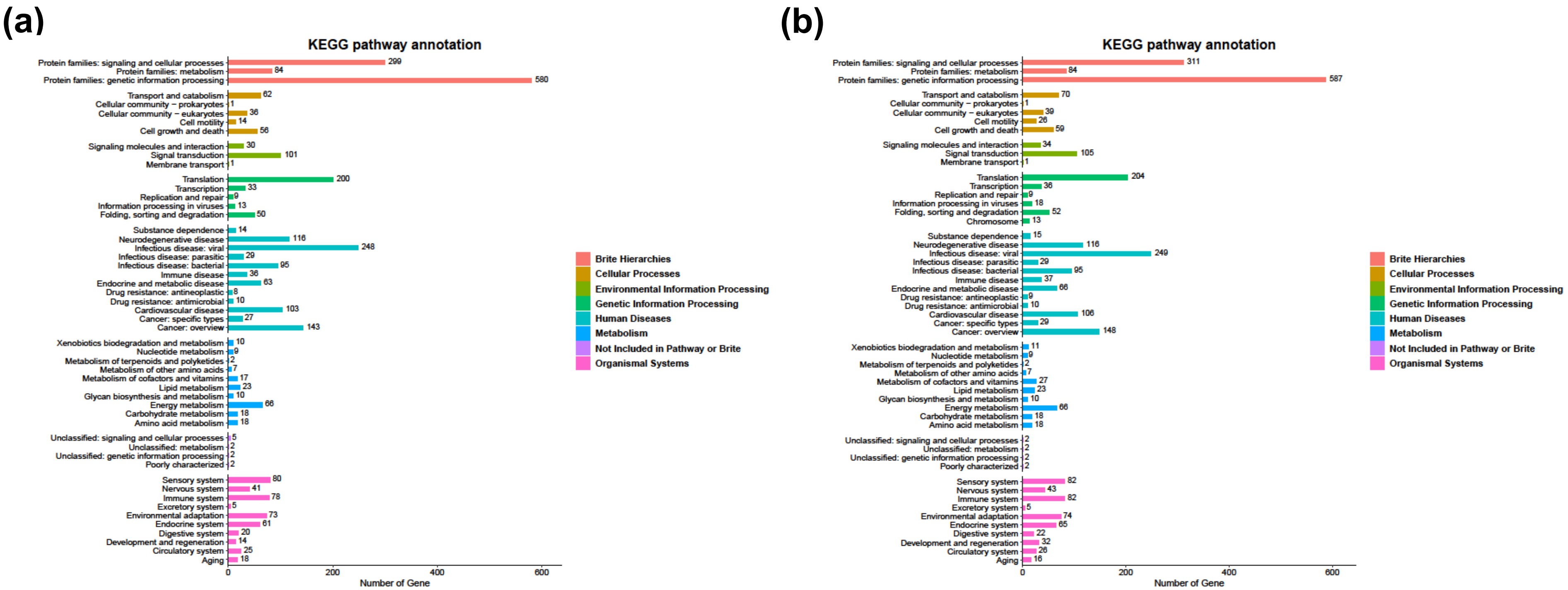
(a)
KEGG pathway annotation
Protein families: signaling and cellular processes 299
Protein families: metabolism 84
Protein families: genetic information processing 580
Transport and catabolism 62
Cellular community − prokaryotes 1
Cellular community − eukaryotes 36
Cell motility 14
Cell growth and death 56
Signaling molecules and interaction 30
Signal transduction 101
Membrane transport 1
Translation 200
Transcription 33
Replication and repair 9
Information processing in viruses 13
Folding, sorting and degradation 50
Substance dependence 14
Neurodegenerative disease 116
Infectious disease: viral 248
Infectious disease: parasitic 29
Infectious disease: bacterial 95
Immune disease 36
Endocrine and metabolic disease 63
Drug resistance: antineoplastic 8
Drug resistance: antimicrobial 10
Cardiovascular disease 103
Cancer: specific types 27
Cancer: overview 143
Xenobiotics biodegradation and metabolism 10
Nucleotide metabolism 9
Metabolism of terpenoids and polyketides 2
Metabolism of other amino acids 7
Metabolism of cofactors and vitamins 17
Lipid metabolism 23
Glycan biosynthesis and metabolism 10
Energy metabolism 66
Carbohydrate metabolism 18
Amino acid metabolism 18
Unclassified: signaling and cellular processes 5
Unclassified: metabolism 2
Unclassified: genetic information processing 2
Poorly characterized 2
Sensory system 80
Nervous system 41
Immune system 78
Excretory system 5
Environmental adaptation 73
Endocrine system 61
Digestive system 20
Development and regeneration 14
Circulatory system 25
Aging 18
0
200
400
600
Number of Gene
Brite Hierarchies
Cellular Processes
Environmental Information Processing
Genetic Information Processing
Human Diseases
Metabolism
Not Included in Pathway or Brite
Organismal Systems
(b)
KEGG pathway annotation
Protein families: signaling and cellular processes 311
Protein families: metabolism 84
Protein families: genetic information processing 587
Transport and catabolism 70
Cellular community − prokaryotes 1
Cellular community − eukaryotes 39
Cell motility 26
Cell growth and death 59
Signaling molecules and interaction 34
Signal transduction 105
Membrane transport 1
Translation 204
Transcription 36
Replication and repair 9
Information processing in viruses 18
Folding, sorting and degradation 52
Chromosome 13
Substance dependence 15
Neurodegenerative disease 116
Infectious disease: viral 249
Infectious disease: parasitic 29
Infectious disease: bacterial 95
Immune disease 37
Endocrine and metabolic disease 66
Drug resistance: antineoplastic 9
Drug resistance: antimicrobial 10
Cardiovascular disease 106
Cancer: specific types 29
Cancer: overview 148
Xenobiotics biodegradation and metabolism 11
Nucleotide metabolism 9
Metabolism of terpenoids and polyketides 2
Metabolism of other amino acids 7
Metabolism of cofactors and vitamins 27
Lipid metabolism 23
Glycan biosynthesis and metabolism 10
Energy metabolism 66
Carbohydrate metabolism 18
Amino acid metabolism 18
Unclassified: signaling and cellular processes 2
Unclassified: metabolism 2
Unclassified: genetic information processing 2
Poorly characterized 2
Sensory system 82
Nervous system 43
Immune system 82
Excretory system 5
Environmental adaptation 74
Endocrine system 65
Digestive system 22
Development and regeneration 32
Circulatory system 26
Aging 16
0
200
400
600
Number of Gene
Brite Hierarchies
Cellular Processes
Environmental Information Processing
Genetic Information Processing
Human Diseases
Metabolism
Not Included in Pathway or Brite
Organismal Systems

Figure S7

(c)

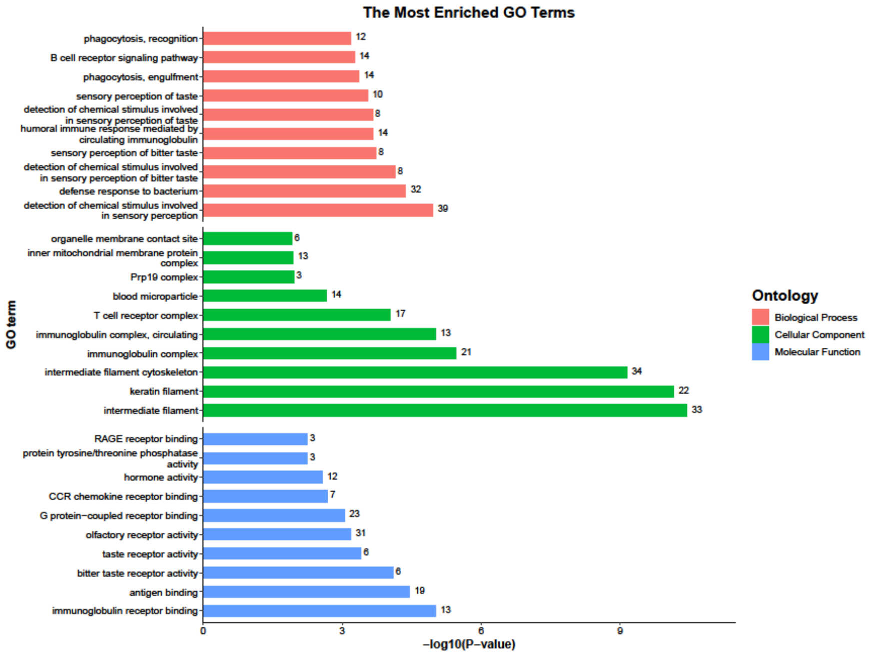
The Most Enriched GO Terms
phagocytosis, recognition
B cell receptor signaling pathway
phagocytosis, engulfment
sensory perception of taste
detection of chemical stimulus involved in sensory perception of taste
humoral immune response mediated by circulating immunoglobulin
sensory perception of bitter taste
detection of chemical stimulus involved in sensory perception of bitter taste
defense response to bacterium
detection of chemical stimulus involved in sensory perception
organelle membrane contact site
inner mitochondrial membrane protein complex
Prp19 complex
blood microparticle
T cell receptor complex
immunoglobulin complex, circulating
immunoglobulin complex
intermediate filament cytoskeleton
keratin filament
intermediate filament
RAGE receptor binding
protein tyrosine/threonine phosphatase activity
hormone activity
CCR chemokine receptor binding
G protein−coupled receptor binding
olfactory receptor activity
taste receptor activity
bitter taste receptor activity
antigen binding
immunoglobulin receptor binding
GO term
−log10(P−value)
Ontology
Biological Process
Cellular Component
Molecular Function

(d)

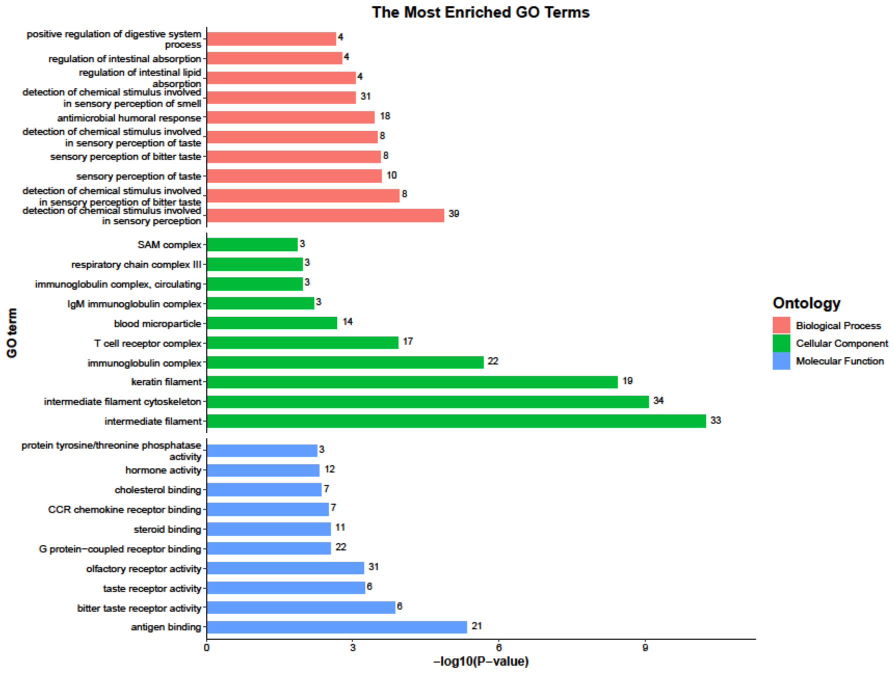
The Most Enriched GO Terms
positive regulation of digestive system process
regulation of intestinal absorption
regulation of intestinal lipid absorption
detection of chemical stimulus involved in sensory perception of smell
antimicrobial humoral response
detection of chemical stimulus involved in sensory perception of taste
sensory perception of bitter taste
sensory perception of taste
detection of chemical stimulus involved in sensory perception of bitter taste
detection of chemical stimulus involved in sensory perception
SAM complex
respiratory chain complex III
immunoglobulin complex, circulating
IgM immunoglobulin complex
blood microparticle
T cell receptor complex
immunoglobulin complex
keratin filament
intermediate filament cytoskeleton
intermediate filament
protein tyrosine/threonine phosphatase activity
hormone activity
cholesterol binding
CCR chemokine receptor binding
steroid binding
G protein−coupled receptor binding
olfactory receptor activity
taste receptor activity
bitter taste receptor activity
antigen binding
GO term
−log10(P−value)
Ontology
Biological Process
Cellular Component
Molecular Function

Figure S7
